# Reliability and readability of five AI chatbots for concussion health advice across retrieval augmented and pretrained models

**DOI:** 10.1038/s41598-026-51281-9

**Published:** 2026-05-03

**Authors:** Hefang Huang, Caihong Zhang, Hao Hu, Xiang Tong

**Affiliations:** 1 Department of Neurosurgery, Honghu People’s Hospital, Honghu, 433200 Hubei China; 2 Department of Nursing, Liyuan Hospital, Tongji Medical College, Huazhong University of Science and Technology, Wuhan, 430077 Hubei China

**Keywords:** Health literacy, Artificial intelligence, Concussion, Large language models, Chatbot, Patient education, Computational biology and bioinformatics, Engineering, Health care, Mathematics and computing, Medical research, Neuroscience

## Abstract

Generative AI is rapidly entering patient education workflows, yet its safety profile for concussion management remains undefined. Utilizing the CHART framework, this cross-sectional audit assessed five platforms, specifically isolating retrieval-augmented generation (RAG) architectures against standard pre-trained Large Language Models (LLMs). We extracted 11 high-volume patient queries from Google Trends and administered them via a zero-shot protocol. Two blinded neurosurgeons then scored the outputs against the 2023 Amsterdam Consensus Statement using four validated instruments: DISCERN and EQIP to evaluate treatment and information quality, GQS for global content quality, and JAMA benchmarks for transparency. Reliability metrics diverged significantly across models (DISCERN and EQIP, *p* < 0.001). Perplexity Pro secured the highest DISCERN (47.36 ± 4.84) and EQIP (65.00 ± 5.48) values, statistically surpassing foundational models like ChatGPT and Gemini (*p* < 0.01) — a performance gap likely driven by its RAG design. In contrast, GQS scores did not differ significantly across models (*p* = 0.373), and JAMA-based transparency remained uniformly low (*p* < 0.001). Readability was assessed using six standard indices (FRES, FKGL, GFI, CLI, ARI, and SMOG), revealing that all models exceeded the 6th-grade reading level; most surpassed 10th-grade, with Perplexity Pro lowest at FKGL = 7.46. Although retrieval-augmented systems improve clinical accuracy, current iterations fail to provide transparent or readable advice. Clinical integration therefore requires rigorous human-in-the-loop verification and a shift toward plain-language algorithm optimization.

**Supplementary Information:**

The online version contains supplementary material available at 10.1038/s41598-026-51281-9.

## Introduction

Clinically synonymous with mild traumatic brain injury (mTBI), concussion stems from biomechanical forces—whether direct or indirect—that precipitate transient neurological dysfunction. While the “mild” nomenclature implies a benign trajectory, the clinical reality is far more complex^1–3^. Concussion is a common form of mild traumatic brain injury that occurs in both sport-related and non-sport-related settings^2,4^. The Global Burden of Disease 2021 study estimated approximately 20.8 million incident traumatic brain injury cases worldwide in 2021, highlighting the substantial public health burden of these injuries^5^. Even though acute recovery normally takes place within weeks, a large group (15–30%) develop Persisting Symptoms after Concussion (PSaC)^6^, which range from vestibulocochlear deficits to mood instability^7–9^. Moreover, in risk populations—in particular, athletes and military personnel—cumulative trauma is getting strongly correlated with neurodegenerative sequelae, including chronic traumatic encephalopathy (CTE)^10,11^. In this way, this mismatch between the seemingly “mild”label and the potential for prolonged disability underscores the need for accurate patient education.

Anxiety often drives patients to skip clinical consultation in favor of web searches. There, they face an “infodemic”—a chaotic mix of conflicting opinions and disjointed data^12,13^. Generative AI has become one of the possible answers to this information gap^14^. Large Language Models (LLMs) theoretically function as information equalizers, distilling dense medical terminology into conversational guidance. However, they suffer from a distinct flaw: “hallucinations,” or the generation of factually incorrect content^15^. A basic conflict between information and medical truth is the danger that an AI may produce convincingly and potentially dangerous advice.

Although large language models are increasingly studied in medical education and patient education, rigorous audits of patient-facing advice remain limited^16,17^. In concussion specifically, online patient materials have long shown important quality and readability deficiencies^18–20^. We suggest that conditions with more complex management, such as concussion, need further examination. To address this, we employed a rigid PICO framework to stress-test five leading LLMs—including GPT-5 and Perplexity Pro—against the 2023 Amsterdam Consensus Statement. Our primary objective was to determine if these tools function as reliable clinical adjuncts or potential vectors of harm, benchmarking them specifically through the lens of patient safety, readability, and actionability.

## Results

### Reliability analysis

We evaluated inter-rater reliability for all scoring systems. Agreement between the two primary raters was good to high: DISCERN, ICC = 0.87 (95% CI, 0.80–0.92); EQIP, ICC = 0.84 (95% CI, 0.77–0.90); GQS, ICC = 0.81 (95% CI, 0.73–0.88); JAMA, ICC = 0.79 (95% CI, 0.70–0.87). These values indicate that the scoring was stable across raters and reproducible.

**Fig. 1:**
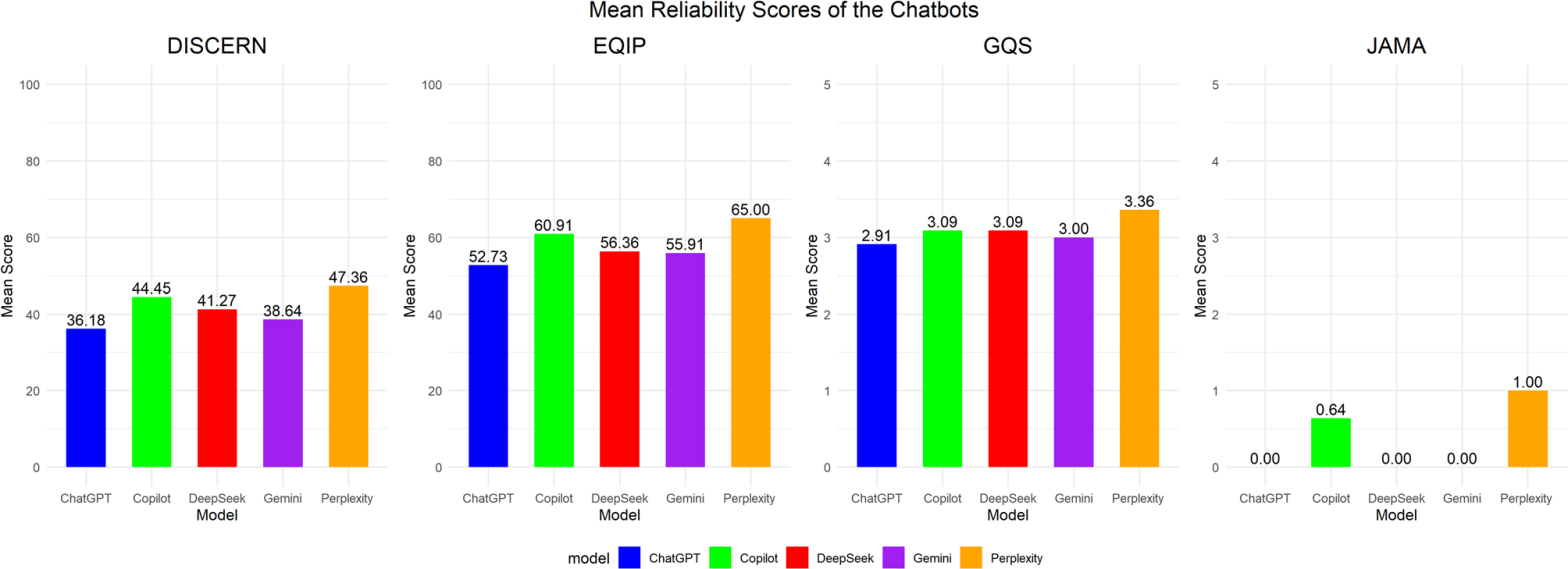
Reliability scores of the chatbots (DISCERN, EQIP, GQS, JAMA).

Table 1 summarizes the descriptive statistics (mean ± SD) for the four quality domains. Figure 1 shows the consistent ranking pattern: Perplexity Pro and Microsoft Copilot formed the highest-performing group for DISCERN and EQIP, ChatGPT and Gemini scored lower, and DeepSeek fell in between them. Kruskal–Wallis testing identified significant between-model differences for DISCERN (*H* = 20.161, *p* = 0.0005, η^2^ = 0.323), EQIP (*H* = 20.432, *p* = 0.0004, η^2^ = 0.329), and JAMA (*H* = 40.350, *p* < 0.001, η^2^ = 0.727). GQS did not differ significantly across models (*H* = 4.249, *p* = 0.3733,η^2^ = 0.005). Effect sizes were large for DISCERN, EQIP, and JAMA; for GQS, they were negligible.

**Fig. 2:**
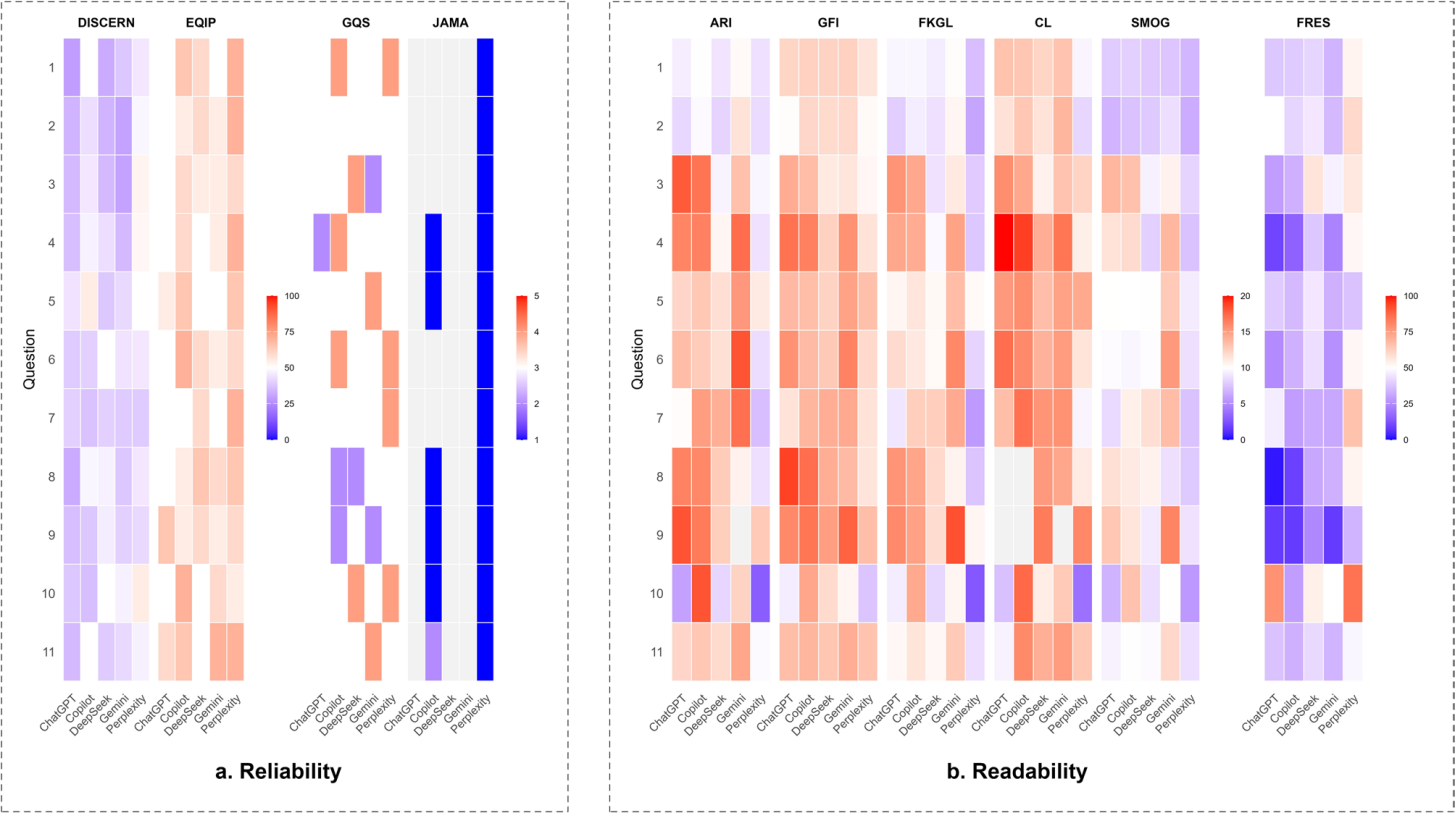
Reliability and readability scores of each question across chatbot models.

Dunn post hoc testing (Table 2) showed that Perplexity Pro scored significantly higher than ChatGPT and Gemini on both DISCERN and EQIP (*r* = 0.545–0.638), indicating large effect sizes for these comparisons. Copilot did not differ significantly from Perplexity Pro on either measure (*r* = 0.030–0.036), which places these two retrieval-augmented systems in a comparable range for those domains. DeepSeek remained intermediate(*r* = 0.115–0.290). ChatGPT and Gemini occupied the lower end of the reliability range. Figure 2 showed the same distribution, with higher values concentrated in the retrieval-augmented systems, particularly for treatment quality (DISCERN) and information quality (EQIP). These results are best interpreted at the domain level, rather than as evidence that one model outperformed all others across every reliability measure.

**Fig. 3:**
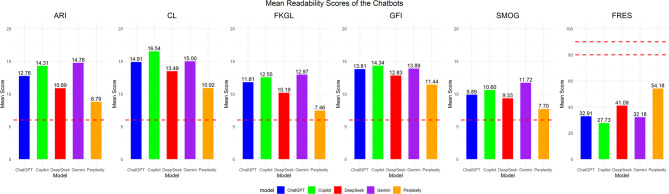
Readability scores of the chatbots (ARI, FRES, GFI, FKGL, CL, SMOG).

**Table 1:** Reliability and Model Comparison Scores Across AI Chatbots (mean ± SD, H, *p*,η^2^) .

	DISCERN	EQIP	GQS	JAMA
ChatGPT	36.18 ± 4.05	52.73 ± 5.18	2.91 ± 0.30	0.00 ± 0.00
Copilot	44.45 ± 6.19	60.91 ± 6.64	3.09 ± 0.70	0.64 ± 0.67
DeepSeek	41.27 ± 6.37	56.36 ± 5.52	3.09 ± 0.54	0.00 ± 0.00
Gemini	38.64 ± 5.35	55.91 ± 5.84	3.00 ± 0.63	0.00 ± 0.00
Perplexity	47.36 ± 4.84	65.00 ± 5.48	3.36 ± 0.50	1.00 ± 0.00
*H*	20.161	20.432	4.249	40.350
*p*	0.0005	0.0004	0.3733	0
η^2^	0.323	0.329	0.005	0.727

**Table 2:** Dunn-Test Results for Reliability Scores (p-values and effect sizes r).

	DISCERN	EQIP	GQS	JAMA
ChatGPT–Copilot	0.009(0.404)	0.014(0.386)	0.800(0.027)	0.008(0.381)
ChatGPT–DeepSeek	0.097(0.255)	0.234(0.188)	0.704(0.022)	1(0.0)
Copilot– DeepSeek	0.388(0.148)	0.237(0.198)	0.970(0.006)	0.010(0.381)
ChatGPT–Gemini	0.332(0.140)	0.261(0.170)	0.941(0.012)	1(0.0)
Copilot–Gemini	0.102(0.263)	0.190(0.172)	0.861(0.011)	0.012(0.381)
DeepSeek–Gemini	0.394(0.115)	0.838(0.006)	0.800(0.010)	1(0.0)
ChatGPT–Perplexity	0.001(0.545)	0.000(0.549)	0.500(0.057)	0(0.663)
Copilot–Perplexity	0.369(0.030)	0.261(0.036)	0.656(0.032)	0.052(0.282)
DeepSeek–Perplexity	0.800(0.290)	0.016(0.078)	0.817(0.034)	0(0.582)
Gemini–Perplexity	0.014(0.317)	0.017(0.084)	0.640(0.044)	0(0.582)

###  Readability analysis

**Fig. 4:**
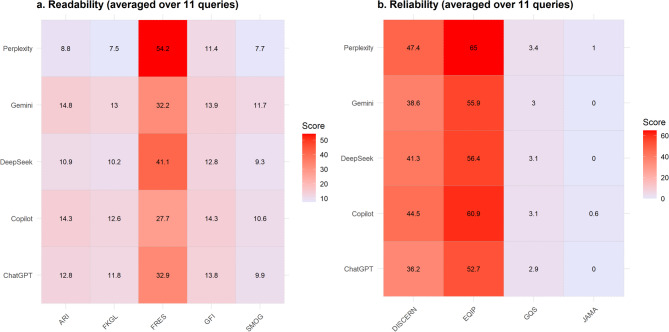
Average readability and reliability scores across all 11 queries for each chatbot model.

The overall results of the readability are provided in Table 3; Fig. 3 when six known indices (ARI, FRES, GFI, FKGL, CL and SMOG) are considered. The overall result was the noncompliance of all five AI platforms: none of them achieved the recommended 6th grade reading level (AMA/CDC standard) in all measures. This means that patient groups having poor health literacy have a systemic obstacle to understanding. Although the benchmarks were not achieved in general, there were still several levels of performance that appeared (as illustrated in Fig. 2 heatmap): Perplexity Pro always made the most accessible content. It had the greatest values of Flesch Reading Ease (FRES), and the lowest estimates at grade level, which is reflected on the heatmap by high-intensity clusters of good metrics. Conversely, Microsoft Copilot and Gemini had the densest syntactically. These models had the lowest readability scores with the complicated sentence constructions that propelled the grade-level estimates considerably high. ChatGPT and DeepSeek were in the middle between moderate complexity and still could not reach the limit of patient-friendly. Even the best performing models, however, were not able to meet clinical utility. Responses to all chatbots were precipitously lower than the desired range of 80–90 (which is usually demanded by plain English medical text). Both FKGL and GFI indices maintained the content at both high-school to collegiate reading level (> 10th grade). Figure 4 shows the average readability and reliability scores across all 11 queries for each chatbot model, providing a summarized view of performance trends in conjunction with the detailed heatmap presented in Fig. 2. This systematic underperformance of the literacy standard highlights one key safety concern: although retrieval-augmented models such as Perplexity Pro achieve slightly high levels of clarity, existing AI-generated advice on concussions is simply unavailable to the masses.

**Table 3:** Readability scores across AI chatbots(mean ± SD).

Program	ARI	FRES	GFI	FKGL	CL	SMOG
ChatGPT	12.76 ± 4.10	32.91 ± 21.81	13.81 ± 2.96	11.81 ± 3.01	14.91 ± 4.45	9.89 ± 2.36
Copilot	14.31 ± 2.88	27.73 ± 11.49	14.34 ± 1.75	12.55 ± 2.11	16.54 ± 2.54	10.60 ± 1.87
DeepSeek	10.89 ± 1.98	41.09 ± 9.70	12.83 ± 1.18	10.19 ± 1.44	13.49 ± 1.93	9.33 ± 1.27
Gemini	14.78 ± 3.63	32.18 ± 11.23	13.89 ± 2.20	12.97 ± 2.71	15.00 ± 2.14	11.72 ± 2.86
Perplexity	8.79 ± 2.43	54.18 ± 13.71	11.44 ± 1.68	7.46 ± 2.14	10.92 ± 2.24	7.70 ± 1.10
6th grade level score	6	80–90	6	6	6	6

## Discussion

Our data reveal a clear architectural divergence in AI performance, consistent with recent comparative audits^21,22^. Retrieval-augmented systems (Perplexity Pro, Microsoft Copilot) achieved higher scores than some standalone LLMs (ChatGPT, Gemini) on DISCERN and EQIP (*p* < 0.001, *r* = 0.545–0.638, η²= 0.32–0.35), while no significant differences were observed for GQS(*r* = 0.006–0.057, η²= 0.001–0.003). These results indicate that, within specific reliability domains, RAG architectures may offer measurable advantages. By querying real-time external databases, RAG models access up-to-date information, unlike foundational LLMs that rely solely on pre-trained parameters with fixed knowledge cutoffs. This feature is particularly relevant in concussion management, where clinical guidelines evolve rapidly—for example, the shift from strict rest to active recovery in the 2023 Amsterdam Consensus. Access to current guidance allows RAG systems to reduce the risk of generating outdated or inaccurate advice.The observed differences were effectively captured by DISCERN and EQIP, instruments originally designed for evaluating static web content. Their ability to reflect enhanced fidelity underscores that RAG architectures can improve information accuracy in domains requiring timely clinical updates. However, these findings should be interpreted by domain rather than as evidence of uniform superiority across all quality metrics. Instead, they suggest that retrieval-augmented systems may be particularly suited for tasks demanding real-time accuracy, adaptability, and the integration of evolving clinical standards. Findings from other medical specialties likewise suggest that chatbot performance is task- and domain-dependent, and should be interpreted relative to evaluation context^21^.

DeepSeek occupied an intermediate position between foundational LLMs and RAG-based models, achieving moderate reliability(*r* = 0.115–0.290) and slightly better linguistic accessibility than ChatGPT. This performance is especially salient since the development of the model is oriented towards non-Western datasets and, consequently, it is more likely to have high cross-linguistic semantic transfer rates in the specialized lexicon of neurotrauma.Nonetheless, the fact that the gap between DeepSeek and the RAG-based in terms of reliability remains unchanged(*r* = 0.458–0.639) indicates that the Corpus Density Hypothesis holds true. DeepSeek’s training corpus likely contains a lower density of Western-specific consensus statements (e.g., CDC or SCAT6 protocols). This observation underscores a broader issue of “algorithmic geography”. If training data lacks regional diversity, AI models may fail to capture the nuance required for global health recommendations, potentially introducing synthesis bias across different populations; medical AI internationalizing, the bias in training data, on a regional basis, may undermine the subtlety and cultural suitability of clinical guidance, introducing unevenness in the synthesis of global health recommendations on different populations.

Our discovery characterizes the emerging capabilities of the medical AI. To start with, the reliability gap established with conventional LLMs (η² = 0.32–0.35) requires a technological shift towards so-called “Human-in-the-Loop” (HITL) designs. Newer versions should not just be passive generation, but instead active clinical verification; that is, “real-time” does not mean “fast,” but means “checked out to be correct, not mere old-fashioned” like that which had previously been tested and found correct. An important frontier to investigate in the future is the behavioral aspect: we have to question how algorithmic advice actually attenuates patient anxiety and decision-making at acute stage of neurotrauma. Will falsely confident AI assure malignant intracranial hemorrhage patients? Lastly, in order to establish AI as a legitimate clinical adjunct, the community needs to shift its solidarity towards a longitudinal clinical metrics (such as DISCERN). It is only through these strict prospective studies that it can be conclusively established whether these tools can play the role of safe symbiotic companions which aid and not interfere with the traditional continuum of concussion care.

We hypothesize that clinically, fidelity becomes functional, hence, communicability is absent. Consequently, it was equally important to find out the linguistic accessibility of created advice as to check its correctness. In order to strictly measure this, we implemented a multi-metric battery consisting of FRES, FKGL, GFI, CLI, and SMOG. Although the use of an indicator based on Flesch is common in the literature, the use of the SMOG index was specifically important to our study in the form of methodological triangulation and this indicates that our measurement of syntactic complexity was taken into holistic complexity and not merely as an outcome of a superficial reading style.

The finding that all models have failed to reach the 6th-grade literacy threshold supports an emerging literature^23^ of a need to address the issue of a complex trap when it comes to health communications in AI. But we believe that these higher grade levels are more or less a creation of algorithmic inflexibility, and not a real representation of syntactic challenge. Traditional formulae like SMOG and FKGL impose severe penalties for polysyllabic words, artificially inflating scores based on word length rather than conceptual difficulty. Challenging nomenclature, including notions like “unconsciousness” or “disorientation” or “rehabilitation” is polysyllabic, but conceptual to the layman. In turn, these static measures, when applied to the conversational, explanatory output of an LLM, are probably associated with an inflationary bias in overestimating actually necessary cognitive load to be used to comprehend the output. This implies that new generation readability tools that are specifically optimized to switchable, conversational AI interfaces are urgently required.

Our qualitative analysis has found out certain lexical obstacles to accessibility. Often, the algorithms reverted to clinical nomenclature e.g. “neurocognitive testing” or “graded return-to-play protocol” that is a source of health literacy friction. In a bid to fight this, subsequent prompt engineering should focus on semantic simplification. As an example, abstract concepts, such as neurocognitive testing, are to be rendered to physical behavior, such as simple thinking-and-memory check. Likewise, graded return-to-play should be more practical presented in terms of step by step return under medical care.Syntactic representation is also in need of compression. We found that important safety messages were usually hidden in twisted sentence forms of declaration. These need to be substituted with imperative-based syntax (buttons) in order to decrease the cognitive load. The result should cause immediate action: e.g. seek urgent care in case of vomiting or confusion, but not an explanation that these symptoms may be bound to a complication.

Existing algorithmic outputs are rich in information, but their syntactic content is usually laid down in a prohibitive manner. This complication leads to the existence of a literacy barrier among non-medical stakeholders—a concern also raised in studies on sensitive health topics like STDs^24^, particularly adolescents, parents, and sideline coaching staff, who are required to resolve the key decisions in the pressure situation.To solve this issue, further prompt engineering needs to focus on the information scaffolding. Instead of large narrative blocks, critical clinical data, including warning signs on red flags and reentry pathways, are needed to be presented through plain language architectures^25^. This involves strict application of the terse syntax, bulletted schemas and bolded imperatives to keep the maximum actionability and minimum linguistic length on users with different health literacy levels.

Since our results revealed that existing models already overestimate patient literacy levels in a systematic way—mirroring a long-standing trend in traditional patient education materials^26^, the next stage in the future development of the field should focus on the fine-tuning of the LLMs specifically on the medical corpora in plain-language format. Moreover, we support the idea of penalizing models because of their jargon density in training, enforcing a default output so that they become legible but actionable. Besides, we support the introduction of Human-in-the-Loop (HITL) architectures. A key application that could be included in the next-generation systems is to dynamically balance the generation pipeline with changing standards (e.g., SCAT6 guidelines) to create a hybrid model capable of balancing AI speed with medical specificity. Future work must determine if radical simplification inadvertently dilutes clinical nuance—a trade-off we term the “precision-comprehension paradox”. After all, the justification of these tools is the shift in a form of textual analysis, to an actual longitudinal investigation of behavior. It is imperative that we ask ourselves in detail, is AI-augmented education in any way physically enhancing patient adherence to return-to-play guidance and fine-tuning long-term recovery curves in various populations.

Several limitations should be noted. First, only 11 patient queries were used, which may limit generalizability across the full spectrum of patient concerns. Second, although DISCERN and EQIP were developed for static web content, they were applied to AI-generated dynamic responses, which may not fully capture quality. Third, using web-based interfaces to maintain ecological validity reduced reproducibility, as opaque default settings replaced hardcoded API hyperparameters. Dual-pass checking improved rating consistency, but the small number of raters (*n* = 2) limited statistical power, and results reflect a performance snapshot rather than long-term consistency. Fourth, Google Trends queries, while broadly representative, may miss local demographic or cultural nuances. Fifth, applying legacy tools such as JAMA may penalize models lacking human-friendly metadata. Sixth, surrogate endpoints like quality and readability metrics do not directly measure patient comprehension or behavioral adherence. Seventh, no external baseline comparison with traditional patient education resources was included. While the 2023 Amsterdam Consensus Statement and CDC guidelines served as internal references, they cannot substitute for practical benchmarks, and future studies should incorporate such baselines to contextualize AI performance. Finally, although a standardized format ensured consistency, the potential sensitivity of different architectures to linguistic variations remains a limitation. Future research should evaluate diverse, conversational patient queries to confirm the generalizability of these findings.

Taken together, these limitations suggest that current AI-generated concussion education should be interpreted with caution and cannot yet serve as an independent patient-facing tool.The existing levels of AI-powered concussion education are characterized by the strong heterogeneity of reliability and systemic inability to reach people with language. Even with the adoption of more advanced architectures, the same deficit applies: as of today, no model is sufficiently safe to engage patients autonomously, needless to say; this demands a reconsideration of the structure not by the bare performance of generation speed, but by the realization of provenance and accessibility by an algorithm. These systems should also not be seen as independent health advisors until the validation protocols are changed to dynamic benchmarks (longitudinal clinical outcome) instead of being applied in an entirely adjunctive role.

## Materials and methods

### Data collection and query selection

We ranked standardized terminology with the Medical Subject Headings (MeSH) database in order to capture the online footprint of the lay population and the digital representation of mild traumatic brain injury (mTBI). Among the search terms used were: concussion, mild traumatic brain injury, brain concussion among others.

To assess global information-seeking behavior, we analyzed Google Trends data over a five-year interval (October 15, 2020—October 15, 2025). Rather than relying on exact keyword matching, we employed the ‘Topic’ feature^27^. This setting aggregates multilingual and synonymous inputs—mapping lay terms such as “head bump” to “concussion”—to capture a broader spectrum of user intent^28^. From the normalized aggregate data, we extracted the top 25 related queries. Subsequent manual curation removed duplicates and non-clinical navigational searches, such as entertainment queries referencing the film Concussion. Supplementary Table S1 details the full list of 25 raw queries alongside specific exclusion criteria. We stratified the final 11 high-volume queries into three clinical domains: Definition & Diagnosis, Management & Protocols, and Prognosis & Complications (Table 4). This categorization ensures the evaluation covers the full trajectory of patient care. These domains also align broadly with prior patient-prioritization work in concussion, in which patients, caregivers, and clinicians identified symptom recognition, recovery, management, and persistent sequelae as major areas of concern^29^. These selections align with established patient-prioritization research, ensuring the queries capture the most clinically significant concerns identified in recent concussion literature.

**Table 4:** Clinical domain stratification of 11 high-volume Google Trends queries.

Clinical Domain	Selected Queries	Rationale
Definition & Diagnosis	1. what is concussion	Targets disease recognition, symptom differentiation, and the criteria required for an initial diagnosis.
	2. Concussion symptoms	
	3. Concussion signs	
	4. Concussion test	
Management & Protocols	5. Concussion treatment	Pertains to acute interventions, standardized clinical algorithms (e.g., return-to-play), and access to specialist care.
	6. Concussion protocol	
	7. Concussion clinic	
Prognosis & Complications	8. How long does concussion last	Examines the expected recovery timeline alongside risks of chronic sequelae or lingering symptoms.
	9. Concussion recovery	
	10. Concussion syndrome	
	11. Post concussion syndrome	

### AI Models and prompting strategy

We developed the prompt set with input from two senior neurosurgeons and one health informatics specialist. We used a zero-shot design to preserve ecological validity and to approximate how patients interact with publicly available chatbots in routine use. To limit architecture-related bias, we kept all prompts short, neutral, and identical across models, with no added instructions, contextual framing, or prompt engineering that could favor either foundational LLMs or retrieval-augmented systems. This standardized format established a neutral baseline to isolate architectural performance. By minimizing prompt engineering, we ensured that observed variations reflected inherent model capabilities rather than prompt-specific optimizations. Each query was entered in plain English as a natural patient-style question, and we submitted the same wording to every model in a new chat session to reduce carryover effects and context drift.(verbatim prompt strings are available in Supplementary Table S2).

In Table 5, we have audited five different conversational agents. We selected our examples so as to have a representative cross-section of the current AI field: Retrieval-Augmented Generation (RAG) systems (Perplexity Pro, Microsoft Copilot ) that synthesize data in real-time via the web, and Foundational LLMs ( ChatGPT-5, Gemini 2.5 Flash, DeepSeek ) that synthesize data by using predetermined internal parameters as the primary priority. It proved critical to include DeepSeek as an open-weights comparator. This enables the performance of proprietary, black-box systems (e.g., OpenAI, Google) in comparison to transparent models that have a possibility of safe, local implementation in healthcare infrastructure.

**Table 5:** Technical Specifications of AI Models.

Model Name	Developer	Architecture Type	Accessibility	Specific Version / Snapshot ID	Knowledge Cut-off Date
ChatGPT	OpenAI	Generative LLM	Closed Source	GPT-5	Aug 2025
Perplexity Pro	Perplexity AI	RAG (Web-connected)	Closed Source	llama-3.1-sonar-large-32k-online	Real-time Web Access
Gemini	Google DeepMind	Generative LLM	Closed Source	gemini-2.5-flash-001	Aug 2025
Copilot	Microsoft	RAG (Web-connected)	Closed Source	Web interface version (accessed Oct 20–27, 2025)	Real-time Web Access
DeepSeek	DeepSeek AI	Generative LLM	Open Weights	deepseek-chat-v3	Sep 2025

The Access protocol and Ecological Validity The interactions were all held between October 20 and October 27, 2025. To closely recapitulate the virtual presence in the US-based patient, the routings were done to go through a server in San Jose, CA through a Virtual Private Network (VPN), being able to access globally identical model versions.

We have purposely used the public web interfaces and not Application Programming Interfaces (APIs). Although APIs provide strong control over hyperparameters (e.g. Temperature part of the interface can be set to 0), they do not mirror the actual interface faced by the laypersons. Our non-use of APIs was a prioritization of ecological validity, which is an aspect of the disastrous nature of patient access, over algorithmic determinism. As a result, it was within the parameters of the dynamic default systems on the platforms. We also admit that this does add some stochasticity, but a dual-pass verification phase was done that verified the high semantic consistency of replicates. All queries were started in a discrete “New Chat” session to avoid context drift.

### Readability and quality evaluation metrics

Readability Analysis: To give quantitative assessment to the linguistic accessibility of the advice generated, we used a set of six well-known algorithms Flesch Reading Ease (FRES), Flesch-Kincaid Grade Level (FKGL), Gunning Fog Index (GFI), Coleman-Liau Index (CLI), Automated Readability Index (ARI), and the SMOG Index. Each readability metric captures distinct aspects of textual complexity. These include sentence length, word length, syllable count, and the proportion of complex or polysyllabic words; by combining these elements, the metrics provide a multi-faceted view of how easily a reader can process the content^30,31^.These metrics were computed using an automated text analysis tool available at https://readabilityformulas.com. The scoring ranges differ across metrics. Flesch Reading Ease Score (FRES) spans 0 to 100, with higher values reflecting simpler, more accessible text. In contrast, Flesch-Kincaid Grade Level (FKGL), Gunning Fog Index (GFI), Coleman-Liau Index (CLI), Automated Readability Index (ARI), and SMOG Index estimate the U.S. school grade level required for comprehension, where lower scores indicate greater readability. The detailed mathematical formulations for these indices have been described previously. This is a multi-metric procedure that guarantees powerful evaluation on the various aspects of sentence form and vocabulary density.

We compared chatbot readability against the 6th-grade benchmark commonly used in health-literacy research and reflected in CDC plain-language guidance^32,33^. For interpretation, we considered a FRES score of 80 or higher as easily understandable by a general patient population^24,34,35^. For the grade-level metrics (FKGL, GFI, CLI, ARI, SMOG), scores of 6 or lower were treated as appropriate for patient comprehension^24,32^, aligning with recommended thresholds for accessible health communication.

Beyond assessing readability, we evaluated the reliability of AI-generated health information using four established instruments in this study: DISCERN, EQIP, JAMA, and GQS. Each tool examines specific dimensions of content quality and has seen widespread application in prior research for evaluating patient-targeted health information^36,37^.

DISCERN: This tool evaluates the quality of health information^38^, with a focus on treatment options, including source reliability, clarity of aims, description of available treatments, and discussion of risks and benefits38. While the DISCERN handbook provides limited guidance on interpreting overall scores, previous literature categorizes the scores as follows: 63–75 points indicate excellent quality, 51–62 points good quality, 39–50 points fair quality, 27–38 points poor quality, and 16–26 points very poor quality^39^.

EQIP (Ensuring Quality Information to patients): This measure aimed at writing clarity and design, thus changing the EQIP scores to percentages. We measured quality in four levels namely excellent (76–100%), adequate (51–75%), poor (26–50%), and very poor (0–25%)^40^.

JAMA Benchmarks: The tool is specifically used to evaluate the visibility of digital health data (authorship, attribution, disclosure, and currency) which gives a score between 0 and 4^41^.

GQS (Global Quality Scale): The Global Quality Scale provides an overall assessment of the quality of health-related information41. It is a 5-point Likert scale designed to evaluate the flow, usability, and practical value of online information, where 1 indicates very poor quality, 2 indicates poor quality, 3 indicates moderate quality, 4 indicates good quality, and 5 indicates excellent quality.

### Evaluation protocol and bias control

To rigorously control for subjective bias, we implemented a two-stage independent review protocol. Two board-certified neurosurgeons blindly assessed all responses using a structured approach. For each query, reference answers were compiled from multiple authoritative sources, including the 2023 Amsterdam Consensus Statement, CDC guidelines, and relevant literature. Each model’s response was scored item by item using standardized tools, including DISCERN, EQIP, GQS, and JAMA. Scoring elements were compared against the reference answers; for example, DISCERN items such as treatment option diversity and information transparency were evaluated relative to these references. This approach produced quantitative results and reflected both reliability and readability.We removed metadata and standardized fonts to obscure the source of each response. Complete blinding was unattainable for RAG models (e.g., Perplexity, Copilot) because inherent features, including inline hyperlinks and distinctive formatting, could reveal the model. Evaluators were directed to judge the responses solely on content, while acknowledging that some residual brand recognition bias might persist.

We quantified inter-rater reliability using the Intraclass Correlation Coefficient (ICC, two-way random, average measures) for all continuous outcomes, including total scores for DISCERN, EQIP, GQS, and JAMA. Discrepancies were resolved through a hierarchical arbitration procedure: Preliminary conflicts were resolved in consensus meetings; the unresolved divergences were resolved by a third senior expert (consultant neurosurgeon with > 20 years of experience), whose decision was the ultimate ground truth in this study.

### Ethics and data use

The Institutional Review Board of Honghu People’s Hospital formally exempted this study protocol from ethical review (Exemption No. HHRY-202566). We relied exclusively on aggregated, publicly accessible data from Google Trends and AI-generated content. No identifiable patient information or human subjects were involved. Since the evaluating neurosurgeons are authors of this manuscript acting in an expert capacity—rather than recruited participants—informed consent was not required. Reporting follows the Chatbot Assessment Reporting Tool (CHART) standards^42,43^.

### Statistical analysis

The data analyses and computational procedures were performed on the RStudio integrated development environment (app version 2024.04.1, posit Software, pBC, Boston, MA), with all the statistical processing being done using the R programming language (version 4.3.3, R Core Team, Vienna, Austria). Quality (DISCERN, EQIP, JAMA, GQS) and readability metrics are provided in the form of means and standard deviations (SD). Before we tested the hypothesis, we determined the properties of data distribution using the Shapiro-Wilk test which showed that the data is not normally distributed among datasets (*p* < 0.05). As a result, we used the Kruskal-Wallis H test that was conducted non-parametrically to ascertain general statistical differences of the five AI models.For Kruskal–Wallis comparisons, effect sizes were calculated using eta-squared (η²). In cases of significant main effects, the post hoc test of Dunn was used to compare means two-by-two, with only the strict use of Bonferroni corrections to control family-wise error rate. Pairwise effect sizes were calculated using r to quantify the magnitude of each difference. Where critical differences were brought, both statistical results were put in a clinical context; in addition to reducing to the raw p-values, we assessed whether mean scores had fallen within the safe zone (≤ 6th-grade level) established by the AMA and CDC. The tests that were used were all in the form of a two-tailed test with the significance level set at α = 0.05. This manuscript underwent English language editing and stylistic refinement using ChatGPT (GPT-5 ; OpenAI) and Gemini (Google). We verified all AI-generated content and accept full responsibility for the final text.

## Supplementary Information

Below is the link to the electronic supplementary material.

Supplementary Material 1

Supplementary Material 2

Supplementary Material 3

## Data Availability

The main text and Supplementary Materials contain all data supporting these findings. Our datasets comprises AI-generated responses to standardized concussion queries, assessed via validated instruments; we did not collect sensitive patient information. The corresponding author will provide further details upon reasonable request.
