# Supplementary material for: Reliability and readability of five AI chatbots for concussion health advice across retrieval augmented and pretrained models
Source: Sci Rep. 2026 May 3;16:20423. doi: 10.1038/s41598-026-51281-9 (PMC13328287; doi:10.1038/s41598-026-51281-9)
Supplement: Supplementary file 2 — Supplementary Material 2 [file 41598_2026_51281_MOESM2_ESM.pdf]

Table S1. Google Trends data of the 25 most significant keywords queried globally for concussion between 2020 and 2025.

**concussion（Worldwide）**	
**TOP**	**Relevance**
**1. concussion symptoms**	100
2. head concussion(removed)	45
**3. concussion signs**	37
4. signs of concussion(removed)	32
**5. what is concussion**	30
6. symptoms of concussion(removed)	26
7. concussion meaning(removed)	22
8. what is a concussion(removed)	22
9. concussion injury(removed)	22
**10. concussion syndrome**	18
**11. concussion treatment**	17
12. signs of a concussion(removed)	17
**13. concussion protocol**	16
**14. concussion test**	16
15. mild concussion(removed)	15
**16. post concussion syndrome**	15
17. symptoms of a concussion(removed)	14
18. concussion movie (removed)	14
19. concussions(removed)	10
20. concussion headache(removed)	10
**21. concussion clinic**	9
**22. how long does concussion last**	7
23. concussion recovery	7
24. tua concussion (removed)	6
25. concussion ICD-10(removed)	6

Table S2. Verbatim prompt strings used for AI model interrogation

NO	question
1	Please answer this question: concussion symptoms, in English
2	Please answer this question: concussion signs, in English
3	Please answer this question: what is concussion， in English
4	Please answer this question: concussion syndrome, in English
5	Please answer this question: concussion treatment, in English
6	Please answer this question: concussion protocol, in English
7	Please answer this question: concussion test, in English
8	Please answer this question: post-concussion syndrome, in English
9	Please answer this question: concussion clinic, in English
10	Please answer this question: how long does concussion last, in English
11	Please answer this question: concussion recovery, in English
