# Supplementary material for: Reliability and readability of five AI chatbots for concussion health advice across retrieval augmented and pretrained models
Source: Sci Rep. 2026 May 3;16:20423. doi: 10.1038/s41598-026-51281-9 (PMC13328287; doi:10.1038/s41598-026-51281-9)
Supplement: Supplementary file 3 — Supplementary Material 3 [file 41598_2026_51281_MOESM3_ESM.docx]

**Supplementary Dataset S2.** Verbatim raw responses generated by five AI chatbots (ChatGPT-5 Turbo, Perplexity Pro, Gemini, Microsoft Copilot, DeepSeek) for the 11 standardized concussion queries. Data collected in October 2025.

| **AI Model** | **Standardized Query** | **Verbatim Response** |
| --- | --- | --- |
| ChatGPT | Q1. what is concussion | A concussion is a type of mild traumatic brain injury (mTBI) that occurs when a blow, jolt, or sudden acceleration-deceleration to the head or body causes the brain to move rapidly inside the skull. This movement can temporarily disrupt normal brain function, affecting how a person thinks, feels, and reacts.  Common symptoms include headache, dizziness, confusion, nausea, balance problems, sensitivity to light or noise, blurred vision, difficulty concentrating, and sometimes loss of consciousness. Symptoms may appear immediately or develop over hours to days after the injury.  Although most concussions resolve within days to weeks with proper rest and gradual return to normal activity, repeated concussions or premature return to play or work can increase the risk of prolonged recovery or more serious complications.  In clinical terms, a concussion involves functional disturbance rather than structural damage, meaning that standard brain imaging (like CT or MRI) typically appears normal. |
| ChatGPT | Q2. concussion symptoms | A **concussion** is a mild traumatic brain injury (mTBI) caused by a blow or jolt to the head or body that disrupts normal brain function. Symptoms can appear immediately or develop over hours to days. They are typically grouped into four main categories:  1. **Physical Symptoms**  Headache or pressure in the head  Nausea or vomiting  Dizziness or balance problems  Blurred or double vision  Sensitivity to light or noise  Fatigue or drowsiness  Temporary loss of consciousness (not always present)  2. **Cognitive (Thinking) Symptoms**  Difficulty concentrating or remembering  Feeling mentally “foggy” or slowed down  Confusion or disorientation  Trouble processing information  3. **Emotional and Behavioral Symptoms**  Irritability or mood swings  Anxiety or depression  Increased emotional sensitivity  Nervousness or restlessness  4. **Sleep-Related Symptoms**  Sleeping more or less than usual  Trouble falling or staying asleep  **Note:** Symptoms vary by individual and may not all appear at once. In children, signs may include excessive crying, changes in eating or sleeping patterns, or loss of interest in favorite activities.  If concussion is suspected, **medical evaluation** is essential, and physical or cognitive rest is recommended until symptoms improve. |
| ChatGPT | Q3. concussion signs | Concussion Signs  Common signs of a concussion include:  Headache or pressure in the head  Nausea or vomiting  Dizziness or balance problems  Blurred or double vision  Sensitivity to light or noise  Confusion or feeling “foggy”  Difficulty concentrating or remembering  Slurred speech  Delayed response to questions  Loss of consciousness (sometimes)  Changes in mood, behavior, or sleep patterns  These signs may appear immediately after injury or develop over several hours. Anyone showing these symptoms after a head injury should seek medical evaluation promptly.  Would you like me to list symptoms separately from observable signs (e.g., by the person vs. by others), as used in clinical guidelines (e.g., CDC or NICE)? |
| ChatGPT | Q4. concussion test | A concussion test is a clinical assessment used to evaluate whether a person has sustained a concussion — a mild traumatic brain injury (mTBI) typically caused by a blow to the head or body that results in rapid brain movement.  Main Purposes  Concussion tests aim to:  Detect signs and symptoms of brain dysfunction  Assess cognitive, neurological, and balance impairments  Guide diagnosis, treatment, and return-to-play or return-to-work decisions  Common Types of Concussion Tests  Symptom Checklists  Patients report symptoms such as headache, dizziness, confusion, nausea, blurred vision, or memory loss (e.g., SCAT6 symptom scale).  Cognitive Tests  Assess memory, attention, processing speed, and reaction time. Examples include:  SCAT6 / Child SCAT6 (Sport Concussion Assessment Tool, 6th edition)  ImPACT (Immediate Post-Concussion Assessment and Cognitive Testing)  CogSport / Axon  King–Devick Test (evaluates eye movement and processing speed)  Balance and Coordination Tests  BESS (Balance Error Scoring System) or force plate balance tests  Tandem gait and coordination tests  Neurological Examination  Conducted by a clinician to assess cranial nerves, reflexes, strength, sensation, and coordination.  Imaging (if indicated)  While CT or MRI scans are usually normal in concussions, they may be used to rule out structural injuries such as bleeding or skull fracture.  When to Use  Concussion tests are typically used:  Immediately after a suspected injury (sideline, emergency room, or clinic)  During follow-up visits to monitor recovery  Before clearance for return to play, school, or work |
| ChatGPT | Q5. concussion treatment | Concussion Treatment  Concussion treatment primarily focuses on symptom management, rest, and gradual return to normal activities. Since a concussion is a mild traumatic brain injury (mTBI), treatment aims to allow the brain to recover while preventing further injury.  1. Initial Management  Physical and Cognitive Rest: Immediately after the injury, patients are advised to rest for 24–48 hours, avoiding physical exertion, screen time, and mentally demanding activities.  Monitoring: During the first 24 hours, it is essential to observe for warning signs such as worsening headache, vomiting, confusion, or loss of consciousness, which may indicate a more severe brain injury.  2. Symptom Management  Headache: Acetaminophen (paracetamol) may be used; nonsteroidal anti-inflammatory drugs (NSAIDs) are usually avoided in the first 24 hours due to bleeding risk.  Sleep and Mood Disturbances: Good sleep hygiene, limited caffeine, and sometimes short-term use of sleep aids may help. Psychological support may be needed for anxiety or depression.  Cognitive Difficulties: Short periods of mental activity can be resumed gradually as symptoms improve.  3. Gradual Return to Activity  A stepwise return-to-play or return-to-work protocol is recommended:  Symptom-limited activity  Light aerobic exercise  Sport-specific exercise or moderate work tasks  Non-contact training or full job duties without risk  Full return to play or work  Each stage typically lasts at least 24 hours. If symptoms return, the person should drop back to the previous stage and rest before progressing again.  4. Multidisciplinary Care  Persistent post-concussion symptoms may require evaluation by:  Neurologists or sports medicine specialists  Neuropsychologists (for cognitive rehabilitation)  Physical therapists (for balance and vestibular dysfunction)  Occupational therapists (for return-to-work strategies)  5. Prevention and Education  Preventive measures include wearing protective gear (helmets, seatbelts), promoting safe sports techniques, and educating patients about the importance of reporting concussive symptoms promptly.  References (examples):  McCrory P, et al. Br J Sports Med. 2017;51(11):838–847. (Consensus Statement on Concussion in Sport, Berlin 2016)  Centers for Disease Control and Prevention (CDC). HEADS UP: Concussion and Mild TBI Guidelines, 2023.  Would you like me to adapt this into a concise academic-style paragraph (about 200 words) suitable for inclusion in a manuscript? |
| ChatGPT | Q6. concussion protocol | A concussion protocol is a standardized medical and management plan designed to guide the assessment, treatment, and return-to-activity process following a suspected or confirmed concussion. It ensures that athletes, patients, or individuals receive consistent and evidence-based care to prevent further injury and promote recovery.  Key Components of a Concussion Protocol  Immediate Evaluation  Removal from play or activity immediately after a suspected concussion.  On-site assessment using tools such as the SCAT6 (Sport Concussion Assessment Tool, 6th edition) or Child SCAT6.  Observation for red-flag symptoms (e.g., loss of consciousness, repeated vomiting, severe headache, neck pain, confusion, or seizures).  Medical Assessment  Conducted by a qualified healthcare professional.  Includes neurological examination, cognitive testing, symptom assessment, balance testing, and sometimes neuroimaging if serious injury is suspected.  Rest and Recovery  Initial 24–48 hours of relative physical and cognitive rest.  Gradual reintroduction of daily activities (school, work) as symptoms improve.  Monitoring for symptom recurrence.  Graduated Return-to-Play (RTP) Protocol  A stepwise progression, typically consisting of 6 stages:  Symptom-limited activity  Light aerobic exercise  Sport-specific exercise  Non-contact training drills  Full-contact practice (after medical clearance)  Return to competition  Each step should take at least 24 hours, and the athlete should remain symptom-free before advancing.  Return-to-Learn (RTL) Protocol  Academic reintegration plan for students recovering from concussion.  Gradual return to cognitive activities (reading, screen use, classroom participation).  Follow-Up and Long-Term Management  Re-evaluation if symptoms persist beyond 10–14 days (adults) or 4 weeks (children).  Consideration of multidisciplinary management (neurology, physiotherapy, psychology).  Example Guidelines  5th International Consensus Statement on Concussion in Sport (Berlin, 2016)  6th International Consensus Statement (Amsterdam, 2022)  CDC HEADS UP Program (United States)  NICE Clinical Guidelines (UK) |
| ChatGPT | Q7. concussion clinic | A concussion clinic is a specialized medical facility that focuses on the evaluation, diagnosis, and management of concussions—mild traumatic brain injuries (mTBIs) that can occur from sports, falls, accidents, or other impacts to the head.  Key Functions  Comprehensive Assessment:  Clinics typically perform neurological examinations, cognitive testing, balance assessments, and symptom evaluations to determine the severity and nature of the concussion.  Multidisciplinary Care:  Concussion clinics often involve a team of specialists such as neurologists, neuropsychologists, physiatrists, physical therapists, occupational therapists, and athletic trainers who collaborate to create individualized treatment plans.  Rehabilitation and Recovery:  They provide targeted rehabilitation for symptoms such as headaches, dizziness, balance problems, vision issues, and cognitive difficulties, and help guide patients through safe return-to-school, work, or sport protocols.  Education and Prevention:  Clinics also educate patients, families, and athletes about concussion prevention, symptom monitoring, and long-term brain health.  Example  A university-affiliated concussion clinic may offer same-day assessments for student athletes, baseline neurocognitive testing before sports seasons, and individualized rehabilitation programs based on return-to-play guidelines (e.g., CDC, NCAA, or NICE protocols). |
| ChatGPT | Q8. how long does concussion last | The duration of a concussion varies considerably depending on several factors (age, previous concussions, severity, etc.), but here are general guidelines:  Many people recover in 7 to 14 days (~1 to 2 weeks).  Most recover within 2 to 4 weeks (~3 to 4 weeks).  Some symptoms can persist for weeks to months, and in rare cases even years.  If symptoms last beyond ~4 weeks (especially in children) or beyond ~3 months (in adults), this may indicate Post‑concussion syndrome.  So — while most people feel back to normal within a few weeks, it’s important to monitor symptoms and seek medical advice if recovery is delayed. |
| ChatGPT | Q9. concussion recovery | Concussion Recovery  Concussion recovery refers to the process of physical and cognitive healing following a mild traumatic brain injury (mTBI). Recovery times vary widely depending on the severity of the injury, age, prior concussion history, and adherence to management guidelines.  1. Typical Recovery Timeline  Most adults recover within 7–14 days, while children and adolescents may take up to 4 weeks. Persistent symptoms beyond these periods are classified as post-concussion syndrome (PCS).  2. Key Recovery Phases  Acute Rest (First 24–48 hours):  Brief physical and cognitive rest is recommended immediately after injury to allow the brain to initiate healing.  Gradual Resumption:  After 1–2 days, patients are encouraged to progressively return to light activity as tolerated, avoiding symptom exacerbation.  Structured Return-to-Play (RTP) and Return-to-Learn (RTL) Protocols:  Recovery protocols follow stepwise progressions—each stage lasting at least 24 hours and advancing only if symptom-free.  3. Common Symptoms During Recovery  Headache, dizziness, fatigue, difficulty concentrating, sleep disturbances, and emotional changes. Symptoms typically improve gradually but may fluctuate.  4. Management Principles  Avoid activities with risk of re-injury until fully recovered.  Manage sleep, stress, and hydration.  Cognitive rehabilitation and vestibular or vision therapy may benefit patients with prolonged symptoms.  Medical follow-up is essential, especially if symptoms persist or worsen.  5. Prognostic Factors  Slower recovery is associated with younger age, female sex, prior concussions, pre-existing mental health issues, and early severe symptom presentation.  References (examples):  McCrory P et al. Br J Sports Med. 2017;51:838–847.  Lumba-Brown A et al. JAMA Pediatr. 2018;172(11):e182847.  Centers for Disease Control and Prevention (CDC). HEADS UP Clinical Guidance, 2023. |
| ChatGPT | Q10. concussion syndrome | Post-concussion syndrome (PCS) refers to a complex set of physical, cognitive, emotional, and behavioral symptoms that persist for weeks, months, or even longer following a concussion or mild traumatic brain injury (mTBI).  Definition  PCS is typically diagnosed when symptoms such as headache, dizziness, fatigue, irritability, difficulty concentrating, memory problems, insomnia, or sensitivity to light and noise continue beyond the expected recovery period—commonly defined as more than 2–4 weeks in adults and 4 weeks in children.  Pathophysiology  The exact mechanism is not fully understood. Proposed explanations include:  Metabolic and neurochemical disturbances following concussion (e.g., altered glucose metabolism, neurotransmitter imbalance).  Structural or microstructural brain injury, detectable through advanced neuroimaging.  Psychological and biopsychosocial factors, including anxiety, depression, and maladaptive coping responses.  Clinical Features  Common symptoms fall into several domains:  Somatic: headache, dizziness, nausea, fatigue, visual disturbances.  Cognitive: poor concentration, memory difficulties, slowed processing speed.  Emotional/Behavioral: irritability, anxiety, depression, emotional lability.  Sleep-related: insomnia, hypersomnia, or disrupted sleep patterns.  Diagnosis  PCS is a clinical diagnosis based on history and symptom persistence after a documented concussion. Diagnostic criteria vary slightly between systems (e.g., ICD-10, DSM-5), but generally require:  A history of head injury with loss or alteration of consciousness.  The presence of three or more characteristic symptoms lasting beyond the acute phase.  No other medical, psychiatric, or substance-related cause explaining the symptoms.  Management  Treatment is symptom-oriented and multidisciplinary, often involving:  Education and reassurance about recovery expectations.  Gradual return to physical and cognitive activity.  Cognitive rehabilitation for attention and memory deficits.  Medication for specific symptoms (e.g., headaches, sleep disturbance, mood).  Psychological support, including cognitive-behavioral therapy (CBT) when indicated.  Prognosis  Most individuals recover fully within weeks, but 10–20% experience prolonged symptoms. Prognosis is influenced by age, sex, previous concussions, psychiatric history, and early symptom severity. |
| ChatGPT | Q11. post concussion syndrome | Concussion syndrome, also known as post-concussion syndrome (PCS), refers to a complex set of symptoms that persist for weeks or months after a mild traumatic brain injury (mTBI) or concussion.  Definition  Concussion syndrome is characterized by the persistence of physical, cognitive, emotional, and sleep-related symptoms beyond the expected recovery period—typically lasting more than 2 weeks in adults or more than 4 weeks in children.  Common Symptoms  Physical: Headache, dizziness, fatigue, nausea, visual disturbances, sensitivity to light or noise.  Cognitive: Difficulty concentrating, memory impairment, slowed thinking, mental fogginess.  Emotional/Behavioral: Irritability, anxiety, depression, emotional lability.  Sleep Disturbances: Insomnia, hypersomnia, or altered sleep patterns.  Pathophysiology  The exact mechanisms remain unclear, but proposed explanations include:  Persistent metabolic or microstructural brain changes after injury.  Dysregulation of autonomic and cerebral blood flow.  Psychological factors such as anxiety or expectation effects.  Diagnosis  Diagnosis is clinical and based on the persistence of symptoms following a concussion, after ruling out structural lesions with neuroimaging. Standardized tools such as the SCAT6, Rivermead Post-Concussion Symptoms Questionnaire, or Neurobehavioral Symptom Inventory may assist in assessment.  Management  Education and reassurance are central.  Gradual return to activity following symptom-limited progression (per recent consensus guidelines, e.g., Amsterdam 2022).  Symptomatic treatment (e.g., for headache, sleep, mood disturbances).  Multidisciplinary rehabilitation, including cognitive therapy, vestibular therapy, and psychological support when indicated.  Prognosis  Most patients recover within 1–3 months, but a minority experience prolonged symptoms lasting several months to a year or more. Risk factors for prolonged PCS include female sex, prior concussion, pre-existing anxiety/depression, and high initial symptom burden. |
| Perplexity | Q1. what is concussion | A concussion is a mild traumatic brain injury (often called a TBI) that occurs when a bump, blow, or jolt to the head—or sometimes to the body—causes the brain to move rapidly back and forth inside the skull. This sudden movement can cause the brain to twist, stretch, or compress, leading to temporary disturbances in brain function such as headaches, dizziness, confusion, and memory problems.[1][2][3]  Medically, a concussion involves a temporary alteration of mental status or consciousness resulting from mechanical force or trauma. It often does not involve a loss of consciousness or visible external injury, but it can still cause significant symptoms and complications. The brain’s movement during a concussion disrupts normal neurotransmission, alters blood flow, and causes metabolic changes that impair brain function temporarily.[3][4][5]  Although concussions are generally considered mild and most people recover fully with proper care, they should be treated seriously. Multiple concussions or inadequate recovery can lead to long-term issues such as chronic headaches, cognitive impairment, or the neurodegenerative condition known as chronic traumatic encephalopathy(CTE).[6]  https://my.clevelandclinic.org/health/diseases/15038-concussion  https://concussionfoundation.org/concussion-resources/what-is-concussion/  https://www.barrowneuro.org/condition/concussion/  https://qbi.uq.edu.au/concussion/what-is-concussion  https://www.aans.org/patients/conditions-treatments/concussion/  https://www.trihealth.com/services/trihealth-orthopedic-and-sports-care/services/sports-medicine/concussion-program/what-is-a-concussion  https://www.mayoclinic.org/diseases-conditions/concussion/symptoms-causes/syc-20355594  https://www.merriam-webster.com/dictionary/concussion  https://myhealth.alberta.ca/Health/pages/conditions.aspx?Hwid=custom.ab_concussion_ac_adult  https://www.upmc.com/services/orthopaedics/conditions/concussion  https://kidshealth.org/en/parents/concussions.html  https://www.cdc.gov/heads-up/about/index.html  https://www.ncbi.nlm.nih.gov/books/NBK537017/  https://www.chop.edu/conditions-diseases/concussion |
| Perplexity | Q2. concussion symptoms | Concussion symptoms can vary widely, appearing immediately or hours to days after a head injury. They are commonly grouped into four categories: physical, cognitive, emotional, and sleep-related symptoms.[1][2][3]  Physical symptoms  Physical signs are often the first noticed:  • Headache or a feeling of pressure in the head[4]  • Nausea or vomiting[4][1]  • Dizziness or balance problems[1]  • Blurry or double vision; sensitivity to light or noise[2][1]  • Fatigue, drowsiness, or low energy[1]  • Ringing in the ears (tinnitus)[5]  • Numbness, weakness, or poor coordination in severe cases[6]  Cognitive symptoms  Concussion often affects thinking and memory abilities:  • Confusion or feeling “foggy”[7][5]  • Trouble concentrating or paying attention[2][1]  • Memory loss, especially about the event causing the injury[7][4]  • Slow thinking or delayed responses[3][2]  • Difficulty learning new information[6]  Emotional and behavioral symptoms  Emotional regulation may shift noticeably:  • Irritability or increased emotionality[8][3]  • Anxiety or nervousness[1]  • Sadness or depression[3][1]  • Restlessness or personality changes[4]  Sleep-related symptoms  Changes to sleep pattern are common in concussion recovery:  • Trouble falling or staying asleep[3][1]  • Sleeping more or less than usual[2][1]  • Feeling unrested after sleep[3]  When to seek medical attention  Immediate medical evaluation is necessary if serious warning signs appear, such as:  • Worsening or persistent headache[6]  • Repeated vomiting[6]  • Slurred speech or difficulty awakening[6]  • Uneven pupil size, seizures, or increasing confusion[6]  These symptoms may differ by age and severity, but medical observation after any head injury is always recommended.[1][3]  1.https://www.cdc.gov/heads-up/signs-symptoms/index.html  2.https://www.franciscanhealth.org/community/blog/concussion-symptoms  3.https://www.barrowneuro.org/condition/concussion/  4.https://www.mayoclinic.org/diseases-conditions/concussion/symptoms-causes/syc-20355594  5.https://qbi.uq.edu.au/concussion/signs-and-symptoms-concussion  6.https://biausa.org/brain-injury/about-brain-injury/what-is-a-brain-injury/concussion-mtbi/common-symptoms-of-concussion  7.https://www.upmc.com/services/orthopaedics/conditions/concussion  8.https://www.henryford.com/Blog/2020/09/Concussions-Symptoms-and-Signs  9.https://www.cdc.gov/heads-up/media/pdfs/schools/TBI_schools_magnet-508-a.pdf  10.https://www.nm.org/conditions-and-care-areas/neurosciences/concussions/symptoms  11.https://www.americanbrainfoundation.org/diseases/concussion/  12.https://baselinetesting.com/concussion-101-guide-symptoms-treatment-rehab/  13.https://my.clevelandclinic.org/health/diseases/15038-concussion  14.https://www.aans.org/patients/conditions-treatments/concussion/  https://www.cdc.gov/traumatic-brain-injury/signs-symptoms/index.html |
| Perplexity | Q3. concussion signs | Common signs of a concussion can be divided into physical, cognitive, emotional, and sleep-related categories. These symptoms may appear right after the injury or develop hours or days later, and their severity can vary greatly between individuals.[1][2][3][4][5][6]  Physical signs  • Headache or a feeling of pressure in the head[3]  • Nausea or vomiting[4][1]  • Dizziness or balance problems[2][4]  • Blurred or double vision[4]  • Sensitivity to light or noise[2][4]  • Fatigue or low energy[1][2]  • Ringing in the ears[3][1]  • Temporary loss of consciousness (though not always present)[6][3]  Cognitive (thinking) signs  • Confusion or disorientation[6][1]  • Slow or delayed response to questions[3][4]  • Trouble concentrating or remembering[2][4]  • Feeling mentally “foggy” or groggy[2][3]  • Forgetting recent events or the injury itself (amnesia)[7][1]  Emotional and behavioral signs  • Irritability or unusual emotional reactions[4][6][2]  • Anxiety, sadness, or depression[4][2]  • Personality changes or inappropriate emotions[6][3]  Sleep-related signs  • Sleeping more or less than usual[2]  • Trouble falling asleep or staying asleep[8][2]  • Feeling unrested after sleep[8]  If symptoms such as persistent headache, repeated vomiting, slurred speech, unequal pupils, or loss of coordination occur, seek emergency medical attention immediately.[8]  https://www.mayoclinic.org/diseases-conditions/concussion/symptoms-causes/syc-20355594  https://www.cdc.gov/heads-up/signs-symptoms/index.html  https://qbi.uq.edu.au/concussion/signs-and-symptoms-concussion  https://www.summahealth.org/orthopedic/patient-education/concussion-management-program/concussion-signs-and-symptoms  https://www.cdc.gov/traumatic-brain-injury/signs-symptoms/index.html  https://ada.com/signs-of-concussion/  https://www.upmc.com/services/orthopaedics/conditions/concussion  https://biausa.org/brain-injury/about-brain-injury/what-is-a-brain-injury/concussion-mtbi/common-symptoms-of-concussion  https://schools.utah.gov/schoolsafetyandstudentservices/pdfs/ConcussionSignsSymptomsChecklist.pdf  https://www.health.harvard.edu/blog/warning-signs-of-a-concussion-2018071714289  https://dph.sc.gov/health-wellness/health-safety/traumatic-brain-injury/symptoms-and-signs-concussion-or-traumatic  https://www.americanbrainfoundation.org/diseases/concussion/  https://qbi.uq.edu.au/brain/concussion/delayed-concussion-symptoms  https://www.deaconess.com/Services/Concussion-Clinic/Concussion-Signs-and-Symptoms  https://my.clevelandclinic.org/health/diseases/15038-concussion  https://www.aans.org/patients/conditions-treatments/concussion/ |
| Perplexity | Q4. concussion test | A concussion test is a medical assessment used to evaluate brain function after a head injury. It typically involves symptom questionnaires and cognitive testing to check memory, attention, reaction time, and physical functions like balance and coordination. The test helps determine if a concussion has occurred and its severity by comparing post-injury results to baseline measurements or average norms.  There are several types of concussion tests:  • Baseline tests measure normal brain function before injury, often done in athletes.  • Post-concussion tests assess brain function after injury to detect impairments.  • Screening tools and questionnaires are used by healthcare professionals to diagnose concussions.  • Some tests are computerized, like the ImPACT test, which evaluates memory, reaction time, and processing speed.  Concussion tests do not typically include brain imaging, as concussions usually do not show up on CT or MRI scans unless there is a more serious injury. Proper diagnosis is important for treatment planning and recovery monitoring.[1][2][3][4][5]  https://my.clevelandclinic.org/health/diagnostics/22267-concussion-test  https://ahs.atlantichealth.org/conditions-treatments/neuroscience/concussions/concussion-testing-screening.html  https://www.mayoclinic.org/tests-procedures/concussion-testing/about/pac-20384683  •https://medlineplus.gov/lab-tests/concussion-tests/  •https://broadviewhealthcentre.com/online-concussion-test/  •https://braintherapyclinic.com/free-online-concussion-test/  •https://www.concussioninsportgroup.com/scat-tools/  https://www.upmc.com/services/orthopaedics/services/sports-medicine/services/concussion/baseline  •https://www.augie.edu/sites/default/files/u54/scat2.pdf  https://www.cognitivefxusa.com/blog/do-i-have-a-concussion-quiz  https://www.youtube.com/watch?v=GDzI5et7lmk  https://my.clevelandclinic.org/health/diseases/15038-concussion  https://www.ausport.gov.au/concussion/medical_practitioners/link_listing/assessment_of_concussion  https://www.loganhigh.org/concussion  https://bjsm.bmj.com/content/bjsports/early/2017/04/26/bjsports-2017-097506SCAT5.full.pdf  https://www.abbott.com/corpnewsroom/diagnostics-testing/concussion-assessment-heres-how-the-process-works.html  https://pmc.ncbi.nlm.nih.gov/articles/PMC9494594/  https://dictionary.cambridge.org/zhs/词典/英语/concussion |
| Perplexity | Q5.concussion treatment | Concussion treatment primarily involves a combination of physical and mental rest followed by gradual reintroduction of activities to aid brain recovery. Initially, relative rest is recommended for the first couple of days after injury, avoiding activities that worsen symptoms such as intense concentration, screen time, or physical exertion. However, complete sensory deprivation or prolonged bed rest is discouraged as it can prolong symptoms and cause mood and physical issues.  After this rest period, light physical activity, such as walking or stationary biking, can help speed recovery if it does not worsen symptoms. Activities should be increased gradually under medical supervision, avoiding any high-risk head impact activities until full recovery. Some cases may require therapies for vision, balance, or cognitive symptoms.  Active rehabilitation is now favored over prolonged rest, with a paced return to normal activities and work or school accommodations as needed. For prolonged symptoms, multi-faceted rehabilitation addressing physical, cognitive, and psychological health may be necessary. Professional evaluation and monitoring are important throughout the recovery process to ensure safe progression and manage any persistent symptoms.[1][2][3][4][5][6]  https://www.mayoclinic.org/diseases-conditions/concussion/diagnosis-treatment/drc-20355600  https://www.cdc.gov/heads-up/guidelines/recovery-from-concussion.html  https://www.brownhealth.org/be-well/concussion-new-approach-treatment  https://www.concussionalliance.org/guidelines-for-recovery  https://www.ncbi.nlm.nih.gov/books/NBK185342/  https://myhealth.alberta.ca/Health/pages/conditions.aspx?Hwid=custom.ab_concussion_ac_adult  https://sportsmedicine.mayoclinic.org/condition/concussion/page/1  https://www.healthdirect.gov.au/concussion  https://www.canada.ca/en/public-health/services/diseases/concussion-sign-symptoms.html  https://my.clevelandclinic.org/health/diseases/15038-concussion  https://uvahealth.com/services/neurology/tbi-concussion  https://www.mayoclinic.org/zh-hans/diseases-conditions/traumatic-brain-injury/diagnosis-treatment/drc-20378561  https://www.redcross.org/take-a-class/resources/learn-first-aid/concussion |
| Perplexity | Q6. concussion protocol | Concussion protocol refers to a set of guidelines and steps designed to manage and treat individuals who have suffered a concussion, which is a type of mild traumatic brain injury caused by a blow or jolt to the head or body resulting in the brain moving rapidly within the skull. The main goals are to ensure proper diagnosis, safe recovery, and prevention of further injury.  Key aspects of a concussion protocol include:  • Immediate removal from activity or play when a concussion is suspected.  • Reporting the injury to medical professionals such as athletic trainers or doctors.  • Medical evaluation which may include neurocognitive testing.  • Rest and avoiding activities that worsen symptoms.  • Gradual return to physical and cognitive activities when symptoms improve, usually in a stepwise manner over several days.  • Medical clearance from a healthcare professional before full return to play or normal activities.  • Monitoring for symptoms such as headache, dizziness, nausea, coordination problems, and cognitive difficulties.  • Avoiding rushing the recovery process and ensuring individualized care because recovery varies for each person.  For athletes, this often involves a "Return to Play" protocol with staged increases in activity such as light aerobic exercise, sports-specific drills without contact, full contact practice, and finally return to competition only after being symptom-free and cleared by a medical provider.  Concussion protocols are established by health authorities, sports organizations, and medical institutions to reduce risks and promote safe recovery following a concussion.[1][2][3][4]  https://www.cdc.gov/heads-up/guidelines/recovery-from-concussion.html  https://www.orlandoortho.com/wp-content/uploads/2024/05/OOC-Concussion-Protocol-FKK-2024-1.pdf  •https://www.verywellhealth.com/concussion-protocol-5195083  •https://www.leanderisd.org/athletics_concussionprotocols/  https://www.cdc.gov/traumaticbraininjury/pdf/tbi_patient_instructions-a.pdf  •http://www.recognizetorecover.org/head-and-brain  https://www.health.mil/News/Dvids-Articles/2023/04/17/news442754?type=Policies  •https://qcctigers.com/sports/2021/10/8/concussion-protocol  https://www.owu.edu/about/offices-services-directory/athletic-training/athletic-training-policies-protocols/concussion-safety-protocol/  https://www.redcross.org/take-a-class/resources/learn-first-aid/concussion  •https://www.xaverian.org/athletics/concussion-management-protocol  https://www.mayoclinic.org/diseases-conditions/concussion/diagnosis-treatment/drc-20355600  https://my.clevelandclinic.org/health/diseases/15038-concussion  https://www.nfl.com/playerhealthandsafety/resources/fact-sheets/nfl-head-neck-and-spine-committee-s-concussion-diagnosis-and-management-protocol  https://myhealth.alberta.ca/Health/pages/conditions.aspx?Hwid=custom.ab_concussion_ac_adult  https://www.op97.org/julian/julian-nurse/concusion-protocol  https://www.cdc.gov/heads-up/guidelines/index.html |
| Perplexity | Q7. concussion clinic | A "concussion clinic" is a specialized medical facility or program focused on the diagnosis, treatment, and rehabilitation of patients who have sustained concussions, which are mild traumatic brain injuries. These clinics use a comprehensive, interdisciplinary approach involving specialists like neurologists, sports medicine doctors, psychologists, physical and occupational therapists, and other healthcare providers. They assess symptoms, evaluate brain function, manage recovery plans, and provide support to help patients safely return to daily activities, sports, and school. Services often include medical exams, cognitive and physical testing, rehabilitation therapies, concussion education, and sometimes mental health management. The goal is to promote optimal recovery and prevent long-term complications from concussions.  In summary, a concussion clinic is a healthcare center that specializes in helping individuals recover from concussions through expert evaluation, treatment, and rehabilitation services.[1][2][3][4][5][6]  https://www.archildrens.org/programs-and-services/concussion-clinic  https://utulsa.edu/academics/health-natural-sciences/academics/departments/communication-sciences-disorders/mary-k-chapman-speech-hearing-clinic/concussion-center/  https://nyulangone.org/care-services/concussion-center  https://www.kennedykrieger.org/patient-care/centers-and-programs/concussion-clinic  https://healthcare.utah.edu/the-scope/health-library/all/2015/12/what-does-concussion-clinic-do  https://www.sralab.org/services/concussion-clinic  https://www.providence.org/services/neuroscience/concussion  https://www.childrenshospital.org/programs/sports-concussion-clinic  https://hollandbloorview.ca/services/programs-services/concussion-centre  https://ahs.atlantichealth.org/conditions-treatments/neuroscience/concussions.html  https://www.nationwidechildrens.org/specialties/concussion-clinic  https://www.rehabwashington.com/programs-services/concussion-clinic/  https://www.uhn.ca/TorontoRehab/Clinics/Hull-Ellis_Concussion_Research |
| Perplexity | Q8. how long does concussion last | A concussion typically lasts from a few days to a few weeks, with most people recovering within about 10 days to 2 weeks. However, recovery time can vary depending on the severity of the injury and individual factors. Some symptoms can persist for months or even longer in cases of post-concussion syndrome. It is crucial to be symptom-free before returning to normal activities, and proper medical care and rest are important for recovery. In some cases, symptoms may last up to a year or more, especially if treatment is delayed or if there are repeated concussions.[1][2][3][4][5]  https://www.healthline.com/health/how-long-do-concussions-last  https://www.nm.org/healthbeat/healthy-tips/how-long-until-i-recover-from-a-concussion  https://www.mayoclinic.org/diseases-conditions/post-concussion-syndrome/symptoms-causes/syc-20353352  https://www.impactphysicaltherapy.com/how-long-does-a-concussion-last/  https://www.nationaldizzyandbalancecenter.com/how-long-does-a-concussion-last/  https://my.clevelandclinic.org/health/diseases/15038-concussion  https://www.headway.org.uk/about-brain-injury/individuals/types-of-brain-injury/concussion/  https://www.cdc.gov/traumatic-brain-injury/response/index.html  https://www.bernsteincenterforvisualperformance.com/blog/how-long-do-the-symptoms-of-a-concussion-last.html  https://www.buzzrx.com/blog/how-long-does-a-concussion-last  https://www.mayoclinic.org/diseases-conditions/concussion/symptoms-causes/syc-20355594  https://www.houstonmethodist.org/blog/articles/2024/oct/how-long-does-a-concussion-last/  https://qbi.uq.edu.au/concussion/how-long-does-concussion-last-long-term-effects  https://www.cognitivefxusa.com/blog/symptoms-of-a-concussion-guide  https://www.hyperbaricmedicalsolutions.com/blog/how-long-does-a-concussion-last |
| Perplexity | Q9. concussion recovery | Concussion recovery typically involves a gradual process of rest followed by a controlled return to regular activities. Most people recover within 2 to 4 weeks. During recovery, initial physical and cognitive rest is important to allow the brain to heal. After symptoms improve and nearly resolve, individuals can progressively resume normal activities, including school, work, and light aerobic exercise, as tolerated without symptom return. Return to sports or strenuous physical activity should be done only with medical approval and follow a stepwise protocol. Some individuals may experience prolonged symptoms, known as post-concussion syndrome, which requires further management and therapy. Support from healthcare providers, family, and friends is crucial in recovery. Early controlled aerobic exercise, even with mild symptom exacerbation, has been shown to improve recovery and help prevent persistent symptoms. Cognitive rest involves limiting activities that require significant brain focus, such as screen time and reading, during the acute phase. The recovery journey varies between individuals, with some recovering quickly and others taking months or longer, especially if complications arise.  Key Points in Concussion Recovery:  Initial rest is essential for the first 24 to 72 hours.  Sleep is important and not harmful during recovery.  Gradual return to regular, non-strenuous activities as symptoms improve.  Monitor for worsening or persistent symptoms and seek medical advice.  Return-to-sport is a careful, multi-step process guided by healthcare professionals.  Active, controlled aerobic exercise is beneficial in recovery.  Support and communication with healthcare providers and loved ones aid recovery.  Post-concussion syndrome may require additional therapy and management.  This approach prioritizes balance between rest and gradual reactivation to optimize brain healing after concussion.[1][2][3][4][5][6]  https://www.cdc.gov/heads-up/guidelines/recovery-from-concussion.html  https://www.moodyneuro.org/what-are-the-6-stages-of-concussion-recovery/  https://pmc.ncbi.nlm.nih.gov/articles/PMC7046089/  https://acsm.org/hot-topic-exercise-rest-concussion-recovery/  https://biausa.org/brain-injury/about-brain-injury/nbiic/how-long-will-it-take-me-to-recover-from-a-concussion  https://www.orlandohealth.com/content-hub/recovering-from-a-concussion-what-to-expect/  https://health.cornell.edu/sites/health/files/pdf-library/Recovering-from-Concussion.pdf  https://stcharleshealthcare.org/services/center-orthopedics-and-neurosurgery/concussion-center/concussion-recovery  https://www.cdc.gov/traumatic-brain-injury/media/pdfs/2024/05/patient_discharge_instructions_ENG-508.pdf  https://www.chrichmond.org/blog/details/clued-in-about-concussion-recovery  https://pmc.ncbi.nlm.nih.gov/articles/PMC4441834/  https://www.northfieldhospital.org/concussion-recovery-after-injury-or-fall  https://healthcare.utah.edu/the-scope/health-library/all/2015/12/concussion-recovery-six-step-process  https://www.cdc.gov/traumatic-brain-injury/response/index.html  https://my.clevelandclinic.org/health/diseases/15038-concussion  https://www.orthohealth.com/sports-concussion/  https://www.chop.edu/health-resources/how-brain-rest-works-concussion-recovery |
| Perplexity | Q10. concussion syndrome | Concussion syndrome, officially known as post-concussion syndrome (PCS), is a condition in which the symptoms of a concussion — a mild traumatic brain injury — persist for weeks, months, or even longer after the initial injury.[1][2][3]  Overview  After a concussion, most people recover within two to six weeks. When symptoms last beyond three months, it is diagnosed as post-concussion syndrome. It can occur regardless of the severity of the initial injury and may affect physical, cognitive, and emotional functions.[3][1]  Symptoms  Common symptoms of post-concussion syndrome include:  Persistent headaches (often migraine-like or tension-type)  Dizziness and fatigue  Difficulty concentrating or remembering things  Mood changes such as anxiety, irritability, or depression  Sensitivity to light and noise  Sleep disturbances (insomnia or hypersomnia)  Blurred vision or ringing in the ears.[4][3]  Causes and Mechanisms  The exact cause of PCS is not fully understood. Some theories suggest altered brain chemistry or microstructural neuron damage. Individuals with prior concussions or mental health conditions (e.g., anxiety, PTSD, migraines) are at higher risk.[5][1]  Diagnosis  There is no single test for PCS. Diagnosis relies on a combination of medical history, neurological exams, and symptom persistence over time. Imaging tests may be used to rule out other conditions, such as skull fractures or bleeding.[1][3]  Treatment  There is no specific cure for PCS. Treatments focus on symptom management and rehabilitation:  Medication for headaches, sleep, or mood symptoms.  Physical therapy for balance, neck pain, or dizziness.  Cognitive therapy for memory, attention, and focus issues.  Psychological support for anxiety or depression.[5][3]  Many individuals experience gradual recovery over several months with a structured, multidisciplinary approach involving neurologists, physical therapists, and psychologists.[2][5]  https://www.uclahealth.org/news/article/long-term-symptoms-indicate-post-concussion-syndrome  https://my.clevelandclinic.org/health/diseases/24900-post-concussion-syndrome  https://www.mayoclinic.org/diseases-conditions/post-concussion-syndrome/symptoms-causes/syc-20353352  https://pmc.ncbi.nlm.nih.gov/articles/PMC6755541/  https://www.franciscanhealth.org/community/blog/post-concussive-syndrome  https://www.mayoclinic.org/zh-hans/diseases-conditions/post-concussion-syndrome/symptoms-causes/syc-20353352  https://www.healthdirect.gov.au/concussion  https://www.mayoclinic.org/diseases-conditions/post-concussion-syndrome/diagnosis-treatment/drc-20353357  https://www.massgeneralbrigham.org/en/about/newsroom/articles/what-is-post-concussion-syndrome  https://www.ncbi.nlm.nih.gov/books/NBK534786/  https://www.boystownhospital.org/knowledge-center/post-concussion-syndrome  https://hartfordhealthcare.org/about-us/news-press/news-detail?articleId=21642&publicid=500  https://pmc.ncbi.nlm.nih.gov/articles/PMC3435903/  https://www.cognitivefxusa.com/blog/symptoms-of-a-concussion-guide  https://my.clevelandclinic.org/health/diseases/15038-concussion  https://zh.wikipedia.org/zh-my/腦震盪症候群?oldformat=true  https://www.dancihu.com/yinghan/c/concussionsyndrome91311.htm  https://zh.wikipedia.org/zh-cn/腦震盪症候群?oldformat=true |
| Perplexity | Q11. post concussion syndrome | Post-concussion syndrome (PCS) is a condition characterized by a set of symptoms that persist for weeks, months, or even longer after a concussion or mild traumatic brain injury. These symptoms commonly include headache, dizziness, cognitive difficulties (such as problems with memory, attention, and concentration), sleep disturbances, mood changes (like irritability, anxiety, and depression), and physical symptoms such as sensitivity to light and noise. PCS can last beyond the normal recovery period, often defined as symptoms continuing for more than three months after the injury. Diagnosis is based on history and reported symptoms since there is no specific test for it. Although there is no targeted cure for PCS, treatment usually involves symptom management with medications, physical therapy, behavioral therapy, and education about expectations for recovery. Some patients benefit from a multidisciplinary rehabilitation approach to address the various symptoms effectively.[1][2][3][4]  •https://en.wikipedia.org/wiki/Post-concussion_syndrome  •https://concussionfoundation.org/pcs-resources/what-is-pcs/  •https://www.webmd.com/brain/post-concussion-syndrome  •https://patient.info/brain-nerves/post-concussion-syndrome  https://www.mayoclinic.org/zh-hans/diseases-conditions/post-concussion-syndrome/symptoms-causes/syc-20353352  https://www.massgeneralbrigham.org/en/about/newsroom/articles/what-is-post-concussion-syndrome  •https://pubmed.ncbi.nlm.nih.gov/30482344/  https://behavemed.com/conditions-treated-new-york-psychologists/head-injury-traumatic-brain-injury-post-concussion-syndrome/  https://www.mayoclinic.org/diseases-conditions/post-concussion-syndrome/symptoms-causes/syc-20353352  •https://www.ncbi.nlm.nih.gov/books/NBK534786/  https://thehartmancenter.com/what-is-post-concussion-syndrome-and-how-is-it-treated/  https://www.dizzinessandheadache.com/symptoms/post-concussion-syndrome.html  https://www.childrenscolorado.org/just-ask-childrens/articles/post-concussive-syndrome/  https://my.clevelandclinic.org/health/diseases/24900-post-concussion-syndrome  https://www.sciencedirect.com/science/article/abs/pii/S0889159115000288  https://www.franciscanhealth.org/community/blog/post-concussive-syndrome  •https://emedicine.medscape.com/article/828904-overview  •https://pubmed.ncbi.nlm.nih.gov/15276952/ |
| DeepSeek | Q1. what is concussion | What is a Concussion?  A concussion is a type of traumatic brain injury (TBI) caused by a bump, blow, or jolt to the head or by a hit to the body that causes the head and brain to move rapidly back and forth. This sudden movement can cause the brain to bounce around or twist inside the skull, stretching and damaging brain cells and creating chemical changes in the brain.  Key Characteristics:  It's a Functional Injury, Not a Structural One: Unlike a bruise or bleed in the brain (which can be seen on imaging tests like an MRI or CT scan), a concussion is an injury to the brain's function. It disrupts the way the brain normally works, but it doesn't typically show up on standard scans.  It's Often Called "mTBI": In medical terms, a concussion is often classified as a mild Traumatic Brain Injury (mTBI). While it's considered "mild" compared to more severe TBIs, the effects can still be serious and should not be taken lightly.  Loss of Consciousness is Not Required: A person does not need to be "knocked out" to have a concussion. In fact, most concussions occur without a loss of consciousness.  Common Causes:  Falls  Sports injuries  Car accidents  Being struck by an object  Common Symptoms:  Symptoms can be physical, cognitive, emotional, and related to sleep. They may appear immediately or be delayed by hours or even days after the injury.  Physical: Headache, nausea, vomiting, dizziness, balance problems, sensitivity to light or noise, blurred vision, feeling sluggish or "in a fog."  Cognitive: Confusion, feeling slowed down, difficulty concentrating or remembering, feeling "not right."  Emotional: Irritability, sadness, nervousness, or more emotional reactions than usual.  Sleep: Sleeping more or less than usual, having trouble falling asleep.  Recovery involves both physical and cognitive rest to allow the brain to heal, followed by a gradual return to normal activities under medical guidance. |
| DeepSeek | 2. concussion symptoms | Post-Concussion Syndrome (PCS)  Definition:  Post-concussion syndrome (PCS) is a complex disorder in which a combination of symptoms — such as headaches and dizziness — persists for weeks, months, or sometimes more than a year after the initial concussion (a mild traumatic brain injury).  Common Symptoms:  Symptoms are varied and can be grouped into three main categories:  Physical:  Headaches (often resembling tension-type or migraine headaches)  Dizziness  Fatigue  Sensitivity to light and noise  Blurred vision  Sleep disturbances  Cognitive:  Difficulty with memory  Problems with concentration  "Brain fog" or feeling mentally slow  Emotional/Behavioral:  Irritability  Anxiety  Depression  Mood swings  Key Points:  There is no specific test for PCS; diagnosis is based on a history of a concussion and the persistence of symptoms.  The exact cause is not fully understood but is thought to involve both structural brain changes and psychological factors.  Most people recover fully, but the timeline varies significantly from person to person.  Treatment is symptomatic and multidisciplinary, often involving rest, gradual return to activity, physical therapy, cognitive therapy, and management of specific issues like headaches or mood disorders. |
| DeepSeek | 3. concussion signs | Of course. Here are the common signs and symptoms of a concussion, presented in English.  A concussion is a mild traumatic brain injury that affects brain function. Effects are usually temporary but can be serious. Signs and symptoms can be subtle and may not appear immediately.  They are generally categorized into four groups:  1. Physical Signs & Symptoms  Headache or a feeling of pressure in the head  Nausea or vomiting  Dizziness or balance problems  Blurred or double vision  Sensitivity to light or noise  Feeling tired, sluggish, drowsy, or having low energy  Ringing in the ears (tinnitus)  Loss of consciousness (this happens in less than 10% of concussions)  2. Cognitive (Thinking) Signs & Symptoms  Feeling mentally "foggy" or slowed down  Difficulty concentrating or remembering  Confusion about recent events or surroundings  Feeling dazed or stunned  Amnesia (forgetting events before the injury or difficulty learning new information)  3. Emotional & Mood Changes  Irritability or unusual grumpiness  Sadness or feeling more emotional than usual  Nervousness or anxiety  Increased emotional reactions  4. Sleep-Related Symptoms  Sleeping more than usual  Sleeping less than usual  Difficulty falling asleep  "Red Flag" Signs (Require Immediate Medical Attention)  These signs in the hours or days after a head injury could indicate a more severe problem. Go to an emergency department immediately if the person shows:  One pupil (the black part in the middle of the eye) larger than the other.  A headache that gets worse and does not go away.  Repeated vomiting or nausea.  Slurred speech.  Weakness, numbness, or decreased coordination.  Convulsions or seizures.  Loss of consciousness (even briefly).  Inability to recognize people or places.  Increasing confusion, restlessness, or agitation.  Unusual behavior.  Drowsiness or inability to wake up.  Important Note: This information is for educational purposes only. If you suspect someone has a concussion, they should stop all activity and be evaluated by a healthcare professional. Do not return to sports or strenuous activity until cleared by a doctor. |
| DeepSeek | 4. concussion test | Concussion Test  A concussion test is a set of assessments used by medical professionals to evaluate brain function following a suspected traumatic brain injury (TBI), such as a concussion. Its primary purpose is to diagnose the injury, determine its severity, and guide decisions about treatment and return to activity.  There is no single "concussion test." Instead, it typically involves a combination of the following:  1. Baseline Testing (Pre-injury)  What it is: A pre-season exam that measures a healthy athlete's normal brain function, including memory, concentration, balance, and reaction time.  Purpose: To provide a comparison point if an injury occurs later.  Common Tools: Computerized neurocognitive tests (like ImPACT®, Axon Sports, Cogstate Sport).  2. Sideline/Initial Assessment (Post-injury)  What it is: A quick evaluation performed immediately after a potential head injury, often on the sports field.  Purpose: To make an initial decision about whether the person should be removed from play.  Common Tools:  SAC (Standardized Assessment of Concussion): Checks orientation, immediate memory, concentration, and delayed recall.  BESS (Balance Error Scoring System): Assesses postural stability and balance.  Maddocks Questions: A set of questions designed to assess orientation and memory related to the game (e.g., "Which team are we playing today?").  3. Comprehensive Evaluation (In the Clinic)  What it is: A detailed assessment conducted by a doctor (e.g., a sports medicine physician or neurologist) in a clinical setting.  Purpose: To make a formal diagnosis, rule out more serious injuries (like a skull fracture or bleeding in the brain), and create a management plan.  Components:  Symptom Checklist: Rating the presence and severity of symptoms like headache, dizziness, nausea, and "brain fog."  Cognitive Assessment: Evaluating memory, learning, concentration, and problem-solving skills.  Neurological Exam: Checking vision, hearing, strength, sensation, coordination, and reflexes.  Balance Assessment: More detailed balance testing.  Imaging (if needed): A CT scan or MRI may be ordered if a more severe brain injury is suspected.  Common Computerized Neurocognitive Tests:  ImPACT® (Immediate Post-Concussion Assessment and Cognitive Testing): One of the most widely used tools, measuring verbal and visual memory, processing speed, and reaction time.  Cogstate Sport / Concussion: A computerized battery that assesses cognitive function quickly.  AXON Sports / C3 Logix: A mobile-based platform that integrates cognitive, balance, and vision tests.  Important Note: Concussion tests are aid tools for healthcare professionals. They are not a substitute for a medical diagnosis. Anyone who suspects they have a concussion should seek evaluation from a qualified healthcare provider. |
| DeepSeek | 5. concussion treatment |  |
| DeepSeek | 6. concussion protocol | Concussion Protocol  A concussion protocol is a standardized, step-by-step plan of action used by medical professionals, sports organizations, schools, and other institutions for the identification, management, and recovery of a person who has sustained or is suspected of having sustained a concussion (a mild traumatic brain injury).  Its primary purpose is to ensure consistent and evidence-based care to protect the individual from further harm and promote a safe recovery.  Key Components of a Typical Concussion Protocol:  1. Recognition and Initial Response (On-Site)  Sideline Assessment: Immediate removal from play/activity if a concussion is suspected. The mantra is "When in doubt, sit them out."  Symptom Evaluation: Using standardized tools like the Sport Concussion Assessment Tool (SCAT-6) or the Child SCAT6 to assess symptoms, cognitive function, and balance.  Red Flags Identification: Checking for signs that require immediate emergency care (e.g., worsening headache, seizure, loss of consciousness, repeated vomiting).  2. Medical Evaluation and Diagnosis  A formal diagnosis must be made by a qualified healthcare professional (e.g., a physician) with experience in managing concussions.  This involves a more thorough neurological examination, assessment of symptoms, and review of cognitive function.  3. Rest and Recovery (Initial Phase)  Physical Rest: Limiting physical activities that exacerbate symptoms (e.g., running, weightlifting, sports).  Cognitive Rest: Limiting activities that require mental effort, such as:  Schoolwork, reading, and exams  Screen time (computers, video games, smartphones)  Loud music and social events  The goal is to allow the brain to heal without overexertion.  4. Graduated Return-to-Play (RTP) Protocol  This is a crucial, step-wise process for athletes to return to their sport safely. Athletes must be symptom-free at each stage before progressing to the next. A typical RTP protocol includes:  Symptom-Limited Activity: Daily activities that do not provoke symptoms.  Light Aerobic Exercise: Walking or stationary cycling at a slow to medium pace. No resistance training.  Sport-Specific Exercise: Running or skating drills. No activities with risk of head impact.  Non-Contact Training Drills: More complex training drills (e.g., passing in football). May start light resistance training.  Full-Contact Practice: After medical clearance, participate in normal training activities.  Return to Play/Game: Full clearance to compete.  Each step should take a minimum of 24 hours. If symptoms return at any stage, the individual must drop back to the previous symptom-free stage and try to progress again after 24 hours.  5. Return-to-Learn (RTL) / Return-to-Work Protocol  Similar to the RTP, this is a structured plan for students and employees to gradually return to their cognitive workloads without worsening symptoms. This may involve:  Shortened school/work days.  Extended time for tests and assignments.  Frequent breaks.  6. Clearance for Full Activity  Final clearance for a full return to sports, school, or work is given by a qualified healthcare professional only after the individual has successfully completed the graduated protocols without any recurring symptoms.  Who Uses Concussion Protocols?  Sports Leagues: The NFL, FIFA, NHL, NCAA, and International Olympic Committee (IOC) all have their own detailed protocols.  Schools and Youth Sports Organizations: To protect student-athletes.  Workplaces: Especially in industries with a risk of head injury (e.g., construction).  Military: For personnel who may experience blast injuries or other head trauma.  In summary, a concussion protocol is a systematic and safety-focused framework designed to guide an individual from the moment of a suspected head injury through to a safe and full return to all aspects of their life. |
| DeepSeek | 7. concussion clinic | Concussion Clinic  A concussion clinic is a specialized healthcare facility or center that focuses on the diagnosis, management, and treatment of concussions (also known as mild Traumatic Brain Injuries or mTBI).  The key aspects of a concussion clinic are:  Multidisciplinary Team: It typically staffs a team of specialists who work together. This team may include:  Neurologists  Sports Medicine Physicians  Neuropsychologists  Physical Therapists  Occupational Therapists  Vestibular Therapists  Optometrists or Vision Therapists  Comprehensive Care: Instead of seeing multiple separate specialists, patients receive coordinated care in one place. The clinic addresses all aspects of concussion recovery, such as:  Physical Symptoms: Headaches, dizziness, balance problems.  Cognitive Symptoms: Difficulty with memory, concentration, and "brain fog."  Emotional Symptoms: Irritability, anxiety, and depression.  Sleep Disturbances.  Individualized Treatment Plans: After a thorough assessment, the clinic creates a personalized rehabilitation plan for each patient. This may include:  Vestibular and balance therapy  Cervical (neck) physiotherapy  Graduated exercise programs  Cognitive rest and gradual return-to-activity protocols  Vision therapy  Guidance on return to learn, work, and sport.  In summary, a concussion clinic provides a centralized, expert approach to help patients safely and effectively recover from a concussion.  Example in a sentence:  "After his head injury, the doctor referred him to a concussion clinic for a full assessment and treatment plan." |
| DeepSeek | 8. how long does concussion last | How Long Does a Concussion Last?  There is no single answer, as recovery time from a concussion varies significantly from person to person. However, most people start to feel better within 1 to 3 weeks. For some, symptoms can resolve in a few days, while for others, they may persist for months.  Here’s a more detailed breakdown:  1. General Recovery Timeline (For Most Adults)  Initial Phase (First 1-3 Days): Symptoms are usually at their worst. Physical and mental rest is crucial.  Short-Term Recovery (1 to 3 Weeks): The majority of people see their symptoms gradually improve and resolve completely during this period.  2. Post-Concussion Syndrome (PCS)  If symptoms last for longer than 3 to 4 weeks, it is often referred to as Post-Concussion Syndrome (PCS). This is more common in certain groups, such as:  Older adults  Females  People with a history of previous concussions  Individuals with a history of migraines, anxiety, or depression  With PCS, symptoms can persist for months, and sometimes even a year or longer. However, even in these cases, people typically continue to improve with proper management.  3. Recovery in Children and Adolescents  Children and teenagers often take longer to recover than adults. It's common for their recovery to take four weeks or more. They require careful management and a gradual return to both school and sports.  What Are the Factors That Influence Recovery?  Severity of the Injury: A more severe initial concussion may lead to a longer recovery.  Previous Concussions: A history of multiple concussions can slow down recovery.  Age: As mentioned, younger people tend to need more recovery time.  Overall Health: Pre-existing conditions can play a role.  Quality of Rest and Rehabilitation: Both physical and cognitive rest immediately after the injury are critical. Pushing yourself too hard too soon can prolong recovery.  What Should You Do?  See a Doctor: This is the most important step. Only a healthcare professional can diagnose a concussion and create a management plan.  Rest Initially: For the first 24-48 hours, limit physical and mental activities (like screen time, reading, and school/work).  Gradual Return to Activity: After the initial rest period, gradually and progressively return to your normal routine under the guidance of your doctor.  When to Seek Immediate Medical Attention:  Go to an emergency room if you or someone else experiences:  A worsening headache  Repeated vomiting  A seizure  Slurred speech  Significant drowsiness or inability to wake up  Weakness or numbness in arms or legs  Loss of consciousness  In summary: While most people recover from a concussion within a few weeks, it is a highly individual process. Proper medical evaluation and following a managed recovery plan are essential for a safe return to normal activities. |
| DeepSeek | 9. concussion recovery | Concussion Recovery: A Comprehensive Guide  Concussion recovery is a process that requires patience, proper management, and a gradual return to activities. The brain needs time to heal after this mild traumatic brain injury (mTBI).  The Golden Rule: Physical and Cognitive Rest (The First 24-48 Hours)  Immediately after a concussion, the brain is in a vulnerable state. The most critical step is to reduce both physical and cognitive demands. This is often called "relative rest."  Physical Rest: Avoid activities that are physically exerting or risk another head impact.  No sports, vigorous exercise, or heavy lifting.  Avoid activities that cause jarring, like jumping or running.  Cognitive Rest: Give your brain a break from mental work.  Limit screen time (phone, computer, TV).  Avoid reading, studying, homework, or demanding work tasks.  Reduce activities that require intense concentration or complex thinking.  Important: Complete, strict rest in a dark room for more than 2 days is no longer recommended, as it can slow recovery. The goal is to find a balance—avoiding activities that worsen symptoms without becoming completely isolated.  Gradual Return to Activity  Once your symptoms have significantly improved at rest, you can begin a step-wise return to your normal routine. The key is to proceed gradually, only moving to the next step if you remain symptom-free.  A typical protocol, such as the one from the Consensus Statement on Concussion in Sport, looks like this:  Symptom-Limited Activity: Daily activities that don't provoke symptoms.  Light Aerobic Exercise: Walking, stationary cycling at a slow pace. Goal is to increase heart rate slightly.  Sport-Specific Exercise: Running or skating drills. No head impact activities.  Non-Contact Training Drills: More complex drills (e.g., passing in football/soccer). May start light resistance training.  Full Contact Practice: After medical clearance, participate in normal training activities.  Return to Sport/Play: Normal game play.  Each step should take at least 24 hours. If symptoms return, drop back to the previous symptom-free step and try again after 24 hours.  Common Symptoms and Management  Physical: Headache, dizziness, nausea, sensitivity to light/noise, fatigue, blurred vision.  Cognitive: Feeling "foggy," difficulty concentrating, memory problems.  Emotional: Irritability, sadness, anxiety, emotional instability.  Sleep: Sleeping more or less than usual, trouble falling asleep.  Management:  Headaches: Over-the-counter pain relievers like acetaminophen (Tylenol) are often preferred. Avoid NSAIDs (like ibuprofen or aspirin) initially unless approved by your doctor, as they can increase bleeding risk.  Dizziness/Balance Issues: A vestibular therapist can provide specific exercises.  Vision Problems: A neuro-optometrist can help.  Recovery Timeline and When to Seek Further Help  Typical Recovery: For most adults (80-90%), symptoms resolve within 2 to 4 weeks. For children and adolescents, recovery can sometimes take longer (up to 4 weeks or more).  Persistent Post-Concussive Symptoms (PPCS): If symptoms last longer than expected (e.g., beyond 4-6 weeks), it is considered prolonged recovery. This requires a multidisciplinary approach, which may include:  Neurologist or Concussion Specialist  Physical Therapy (for neck pain, balance, dizziness)  Occupational Therapy (for return to work/school strategies)  Speech-Language Pathology (for cognitive therapy)  Mental Health Professional (for mood-related symptoms)  Key Takeaways for a Successful Recovery  Listen to Your Body: This is the most important rule. Your symptoms are your guide. If an activity makes them worse, stop and rest.  Sleep is Crucial: Ensure you get plenty of quality sleep, as this is when the brain does most of its healing.  Stay Hydrated and Eat Nutritiously: Proper hydration and a balanced diet support overall brain health.  Avoid Alcohol and Recreational Drugs: These can interfere with recovery, sleep, and medication.  Seek Professional Guidance: Always be under the care of a doctor or healthcare provider who can diagnose the concussion, rule out more serious injury, and guide your recovery plan.  Disclaimer: This information is for educational purposes only. Always consult with a qualified healthcare professional for diagnosis and treatment of a concussion. |
| DeepSeek | 10. concussion syndrome | Of course. Here are the symptoms of a concussion, presented in English.  A concussion is a mild traumatic brain injury that affects brain function. Symptoms can be subtle and may not appear immediately. They are often categorized into four main groups:  1. Physical Symptoms  Headache or a feeling of pressure in the head  Nausea or vomiting  Dizziness or balance problems  Blurred or double vision  Sensitivity to light (photophobia) or noise (phonophobia)  Fatigue, drowsiness, or feeling sluggish  Ringing in the ears (tinnitus)  2. Cognitive (Thinking) Symptoms  Feeling mentally "foggy" or slowed down  Difficulty concentrating or remembering  Confusion  Feeling dazed or stunned  Difficulty following conversations or instructions  3. Emotional & Mood Symptoms  Irritability or unusual anger  Sadness or depression  Nervousness or anxiety  More emotional than usual (mood swings)  4. Sleep-Related Symptoms  Sleeping more than usual  Sleeping less than usual  Difficulty falling asleep  Drowsiness  "Danger Signs" - When to Seek Immediate Medical Attention  The following symptoms are red flags and may indicate a more serious brain injury. Seek emergency care immediately if you or someone else experiences:  A headache that gets worse and does not go away.  Weakness, numbness, or decreased coordination.  Repeated vomiting or nausea.  Slurred speech.  A convulsion or seizure.  Loss of consciousness (even briefly).  Inability to wake up or appearing very drowsy.  One pupil (the black part in the middle of the eye) larger than the other.  Significant confusion, restlessness, or agitation.  Unusual behavior or personality changes.  Recognizing faces or places becomes difficult.  Important Note: Anyone with a suspected concussion should be evaluated by a healthcare professional and should not return to sports or physical activities until cleared by a doctor. Rest, both physical and cognitive, is crucial for recovery. |
| DeepSeek | 11. post concussion syndrome | Post-Concussion Syndrome (PCS)  Post-Concussion Syndrome (PCS) is a complex disorder in which a combination of symptoms — such as headaches and dizziness — persists for weeks, months, or sometimes more than a year after the initial injury that caused a concussion.  A concussion is a mild traumatic brain injury (mTBI). Typically, the symptoms of a concussion resolve within 7 to 14 days. However, when they last longer, the condition is classified as PCS.  Common Symptoms of PCS  The symptoms of PCS can be broadly categorized into three groups: physical, cognitive, and emotional/behavioral.  1. Physical Symptoms:  Headaches: Often described as tension-type or migraine-like headaches.  Dizziness: A persistent sense of spinning or lightheadedness.  Fatigue: Overwhelming tiredness and lack of energy.  Sensitivity to Light (Photophobia) and Noise (Phonophobia): Normal levels of light and sound can feel unbearable.  Sleep Disturbances: Sleeping too much, insomnia, or unrefreshing sleep.  Blurred Vision  Ringing in the Ears (Tinnitus)  2. Cognitive Symptoms:  Memory Problems: Difficulty remembering new information (short-term memory).  Difficulty Concentrating: Easily distracted and unable to focus on tasks.  "Brain Fog": A feeling of being mentally slow, foggy, or "not sharp."  Executive Function Issues: Trouble with planning, organizing, and problem-solving.  3. Emotional & Behavioral Symptoms:  Irritability: Getting easily frustrated or angry.  Anxiety: Feeling nervous, worried, or on edge.  Depression: Persistent sadness, loss of interest in activities.  Emotional Lability: Mood swings and heightened emotional reactions.  Personality Changes  Causes and Risk Factors  The exact cause of PCS is not fully understood. It is likely a combination of:  Physiological factors: Structural and metabolic changes in the brain following the injury.  Psychological factors: The stress of the injury and its symptoms can contribute to conditions like anxiety and depression, which can worsen and prolong PCS.  Risk factors that may increase the likelihood of developing PCS include:  Severe initial concussion.  A history of previous concussions or brain injuries.  Older age.  Being female (studies suggest a higher incidence in women).  Pre-existing conditions like migraines, anxiety, or depression.  Diagnosis and Treatment  There is no single test for PCS. Diagnosis is based on:  A detailed medical history of the injury and the symptoms.  A physical and neurological examination.  Neuropsychological testing to assess cognitive function.  Sometimes, brain imaging (like an MRI) is used to rule out other causes, but it often appears normal in PCS patients.  Treatment is symptom-specific and multidisciplinary:  Rest and Pacing: Initially, both physical and cognitive rest are crucial, followed by a gradual, guided return to activity.  Therapies:  Physical Therapy: For dizziness and balance problems.  Occupational Therapy: To help manage daily tasks and return to work/school.  Vestibular Therapy: Specifically for vertigo and dizziness.  Cognitive Behavioral Therapy (CBT): Highly effective for managing the emotional symptoms, pain, and sleep issues.  Medications: To manage specific symptoms like headaches, depression, anxiety, or sleep disorders.  Outlook (Prognosis)  The prognosis for PCS is generally good. Most people recover completely, but the timeline varies significantly from person to person. Recovery can be a gradual process with ups and downs. Early and targeted intervention is key to a successful recovery.  In summary, Post-Concussion Syndrome is a condition where the symptoms of a concussion persist beyond the expected recovery period, affecting a person's physical, cognitive, and emotional well-being. |
| Gemini | 1. what is concussion | A concussion is a mild traumatic brain injury (TBI) caused by a bump, blow, or jolt to the head, or by a hit to the body that causes the head and brain to move rapidly back and forth.  This sudden movement can cause the brain to bounce or twist inside the skull, leading to chemical changes and sometimes stretching or damaging brain cells.  Key characteristics and common symptoms often include:  • Temporary disturbance of brain function: Effects are often short-term, but can sometimes last days, weeks, or longer.  • Physical symptoms: Headache, dizziness, nausea, vomiting, balance problems, and sensitivity to light or noise.  • Cognitive symptoms: Trouble with concentration, memory, confusion, or "mental fog."  • Emotional/Mood symptoms: Irritability, mood changes, and sadness.  • Sleep symptoms: Sleeping more or less than usual, or difficulty falling asleep.  It's important to note that loss of consciousness does not always occur with a concussion. It is a serious injury that requires medical attention and proper rest for recovery. |
| Gemini | 2. concussion symptoms | Concussion symptoms can vary widely and may affect a person's physical, cognitive, emotional, and sleep-related functions. Symptoms may appear immediately or develop hours to days after the injury.  Here are common concussion symptoms, often categorized:  Physical Symptoms:  • Headache  • Nausea or vomiting (especially early on)  • Dizziness or balance problems  • Vision problems (e.g., blurry or double vision, "seeing stars")  • Sensitivity to light or noise  • Feeling tired, low energy  Thinking and Remembering (Cognitive) Symptoms:  • Confusion or feeling "foggy" or "groggy"  • Feeling slowed down  • Attention or concentration problems  • Trouble thinking clearly  • Problems with memory (short- or long-term)  Social or Emotional Symptoms:  • Irritability or easily angered  • Anxiety or nervousness  • Feeling more emotional  • Sadness  • Changes in personality or behavior  Sleep Symptoms:  • Sleeping more than usual  • Sleeping less than usual  • Trouble falling asleep  Danger Signs (Require immediate emergency medical attention - Call 911 or go to the emergency room):  It's important to seek immediate emergency care if you notice any of the following, as they can indicate a more serious brain injury:  • A headache that gets worse and does not go away  • Repeated nausea or vomiting  • Loss of consciousness (even briefly) or increasing drowsiness/inability to wake up  • Slurred speech, weakness, numbness, or decreased coordination  • Convulsions or seizures  • Unusual behavior, increased confusion, restlessness, or agitation  • One pupil larger than the other or double vision  • Inability to recognize people or places |
| Gemini | 3. concussion signs | The signs of a concussion can vary widely and may appear immediately or hours to days after the injury. They are often categorized into physical, cognitive (thinking/remembering), and emotional/mood changes.  Observed Signs (What others may see):  • Appearing dazed or stunned.  • Confusion about events.  • Forgetting instructions, or asking the same question repeatedly.  • Moving clumsily or struggling with balance (unsteady walking).  • Slow response to questions.  • Loss of consciousness (even briefly, though this does not always happen).  • Changes in behavior, such as unusual irritability or inappropriate emotional responses (e.g., crying without explanation).  Reported Symptoms (What the person with the concussion may feel):  • Physical:  • Headache or a feeling of pressure in the head.  • Nausea or vomiting (especially early on).  • Dizziness or balance problems.  • Sensitivity to light or noise.  • Feeling tired or having low energy.  • Vision problems (e.g., blurry vision, "seeing stars").  • Cognitive/Thinking:  • Feeling "foggy" or "groggy."  • Feeling slowed down.  • Difficulty concentrating or paying attention.  • Trouble with memory (e.g., not remembering events before or after the injury).  • Emotional/Mood:  • Irritability or easily angered.  • Sadness or nervousness.  • Feeling more emotional than usual.  • Anxiety.  It is important to seek immediate medical attention (call emergency services or go to the emergency room) if you notice any "Danger Signs," which can indicate a more serious brain injury. These include:  • A headache that gets worse and does not go away.  • Repeated vomiting or nausea.  • One pupil (the black part in the center of the eye) being larger than the other.  • Drowsiness or inability to wake up.  • Slurred speech, weakness, numbness, or decreased coordination.  • Convulsions or seizures.  • Increasing confusion, restlessness, or agitation. |
| Gemini | 4. concussion test | A "concussion test" (or concussion assessment) is a set of evaluations and tools used by healthcare providers to diagnose a concussion after a head injury and to monitor a person's recovery.  Here are the key aspects of concussion testing:  • Purpose: To determine if a head injury is affecting brain function, even if the person did not lose consciousness. It helps guide decisions about treatment and when it's safe to return to normal activities, especially sports.  • What is typically tested: Concussion tests check various mental and physical abilities, including:  • Cognition: Memory, concentration, problem-solving, and reaction time.  • Neurological Function: Balance, coordination, reflexes, vision, and eye movement.  • Symptoms: Assessing and tracking symptoms like headache, dizziness, nausea, light/noise sensitivity, and emotional changes.  • Components of an Assessment:  • Questionnaires/Symptom Checklists: The injured person (or a witness) answers questions about the injury itself (how it happened, loss of consciousness) and current symptoms.  • Physical/Neurological Exam: A healthcare provider checks physical signs like balance, coordination, reflexes, and eye movement.  • Cognitive Testing: Often done on a computer or paper, these tasks test memory, processing speed, and attention. Examples include standardized tools like SCAT5 (Sport Concussion Assessment Tool 5) and computerized tests like ImPACT (Immediate Post-concussion Assessment and Cognitive Testing).  • Baseline Testing: For athletes, some tests (like ImPACT) are done before the sports season starts to establish a "healthy" brain function baseline, which can then be compared to a post-injury test.  • Imaging: A concussion itself usually does not show up on standard imaging tests like X-rays. CT scans or MRIs may be ordered, but only in specific cases (e.g., severe or worsening symptoms) to check for more serious issues like bleeding or swelling in the brain.  Important Note: A concussion test is a clinical tool used by medical professionals; it is not a substitute for a medical evaluation after a head injury. |
| Gemini | 5. concussion treatment | Concussion treatment primarily focuses on rest and a gradual return to activity. It's important to always seek medical attention for a head injury to get a proper diagnosis and guidance.  Here are the key components of concussion treatment:  1. Relative Rest (Especially in the First 24-48 Hours):  • Physical Rest: Limit strenuous activities, sports, or anything that increases symptoms. Light activity like gentle walking is often encouraged, as complete rest (e.g., lying in a dark room all day) is generally not recommended and can impede recovery.  • Cognitive Rest (Brain Rest): Limit activities that require intense concentration, especially if they worsen symptoms. This may include reducing screen time (phones, computers, TV), reading, texting, or school/work that is mentally demanding.  2. Gradual Return to Activity:  • After the initial period of relative rest, you should gradually increase physical and mental activities as tolerated, at levels that do not cause a major worsening of symptoms.  • Light exercise (like a stationary bike or light jogging) can often be started a couple of days after the injury and has been shown to speed recovery.  • A gradual, supervised return to school or work is typical, often with initial modifications (e.g., reduced workload, rest breaks).  • Crucially, do not return to sports or vigorous physical activities until cleared by a healthcare professional. A slow, step-wise return-to-play protocol is essential to prevent the risk of a second injury.  3. Symptom Management:  • Headaches: Over-the-counter pain relievers like acetaminophen (Tylenol) are often recommended. Consult a doctor before taking NSAIDs (like Ibuprofen or Aspirin) as they can sometimes increase the risk of bleeding.  • Monitoring: Have someone stay with you (or the injured person) for the first 24 hours to monitor for any worsening or severe symptoms (e.g., severe headache, worsening vomiting, slurred speech, confusion), which require immediate emergency care.  • Sleep: Maintain a regular sleep schedule, and it's generally okay to sleep, though severe symptoms require monitoring.  4. Specialized Therapies (If Symptoms Persist):  • If symptoms like headache, dizziness, balance issues, or memory/concentration problems persist for an extended period (weeks to months), a healthcare professional may recommend:  • Physical therapy (for balance or neck issues).  • Vision therapy.  • Rehabilitation for cognitive symptoms.  The most important advice is to follow the specific instructions of your healthcare provider, as recovery is highly individualized. |
| Gemini | 6. concussion protocol | The "concussion protocol" refers to a set of standardized guidelines and procedures designed to ensure that an individual who has sustained or is suspected of having sustained a concussion receives appropriate medical care and a safe, gradual return to their normal activities, especially sports.  Key components of a concussion protocol generally include:  1. Immediate Removal from Activity/Play: If a concussion is suspected, the individual must be immediately removed from the activity (e.g., a sports game or practice) and should not return to play on the same day.  2. Medical Evaluation: A qualified healthcare provider must evaluate the individual to diagnose the concussion and rule out more serious brain injuries.  3. Rest (Physical and Cognitive): Initial treatment focuses on relative physical and mental rest to allow the brain to heal. This involves limiting activities that worsen symptoms, which can include screen time (computer, phone, video games, TV), reading, intense thinking/schoolwork, and vigorous physical exertion. Complete rest in a dark room is typically not recommended; light activity as tolerated is often encouraged after the first 24-48 hours.  4. Symptom Monitoring: The individual is monitored for symptoms (such as headache, dizziness, confusion, nausea, sensitivity to light/noise, etc.) and watched for "danger signs" (worsening headache, seizures, repeated vomiting, etc.) that require immediate emergency care.  5. Graduated Return-to-Activity (RTA) / Return-to-Play (RTP) Plan: Once symptoms have resolved, the individual progresses through a structured, multi-step plan to gradually reintroduce physical and cognitive activities. This is often done in stages (e.g., light aerobic exercise, moderate activity, non-contact drills, full practice) with a period of being symptom-free at each stage before advancing to the next.  6. Medical Clearance: Final clearance from a healthcare provider is required before an athlete can fully return to contact practice and competition.  The exact steps and duration vary depending on the individual, the severity of the concussion, and the specific guidelines of the organization (like a sports league, school, or medical facility). The overarching goal is to prevent a second injury before the first one has healed (known as Second Impact Syndrome, which can be life-threatening) and ensure a full recovery. |
| Gemini | 7. concussion clinic | A concussion clinic is a specialized medical facility or program dedicated to the diagnosis, treatment, and management of concussions, which are a type of mild traumatic brain injury (mTBI).  Here are some key aspects of a concussion clinic:  Multidisciplinary Approach: These clinics often utilize a team of specialists, which can include neurologists, neuropsychologists, physical therapists (especially those specializing in vestibular and balance issues), occupational therapists, sports medicine physicians, and athletic trainers.  Comprehensive Assessment: They perform detailed evaluations, which may include neurological exams, cognitive testing (sometimes computerized neurocognitive testing like ImPACT), and assessment of symptoms such as headaches, dizziness, sleep disturbances, and cognitive or emotional changes.  Individualized Treatment: Treatment plans are tailored to the specific symptoms and needs of each patient. This can involve:  Physical and Cognitive Rest: Guidance on gradually returning to school, work, or sports.  Therapies: Vestibular and visual therapy, sub-threshold exercise rehabilitation, and cognitive-behavioral therapy (CBT).  Symptom Management: Treating associated issues like persistent headaches or sleep problems.  Patient Population: Concussion clinics see a wide range of patients, including athletes (sports-related concussion), as well as individuals who have sustained a concussion from car accidents, falls, or other mechanisms of injury.  Focus: The main goal is to ensure a safe and full recovery, preventing prolonged symptoms (Post-Concussion Syndrome) and guiding patients through the process of safely returning to their daily activities. |
| Gemini | 8. how long does concussion last | The duration of a concussion varies significantly from person to person, but here is a general overview:  • Most Common Recovery Time: For most people, concussion symptoms like headache, dizziness, and difficulty concentrating will last 10 to 14 days.  • A Few Weeks: Many people recover fully within a few weeks.  • Prolonged Symptoms: Symptoms can last for days, weeks, or even longer.  • Post-Concussion Syndrome (PCS): A small number of people may experience symptoms that persist for three months or longer, sometimes even up to a year or more. When symptoms last longer than expected (often beyond 3 months, though sometimes defined as more than 28 days), it is referred to as Persistent Post-Concussive Symptoms or Post-Concussion Syndrome.  Key factors influencing recovery time include:  • Severity of the initial injury.  • Age (younger children and older adults may take longer).  • Whether the person has a history of previous concussions.  • Early rest and gradual return to activities (following a doctor's guidance) can help shorten recovery time. |
| Gemini | 9. concussion recovery | Concussion recovery is a process that varies significantly from person to person. While most people recover within a few days or weeks, some may experience symptoms for longer. Medical guidance is essential for safe recovery.  Here are the key aspects of concussion recovery:  1. Relative Rest (Especially in the First 24-48 Hours):  • Initial Focus: The first couple of days often require "relative rest," meaning limiting activities that require significant physical or mental exertion, as symptoms are typically most severe during this time.  • Avoid "Cocooning": Complete rest in a dark room is no longer recommended. Instead, engage in light activities of daily living as tolerated.  • Limit Exertion: Reduce activities that exacerbate symptoms, such as vigorous physical activity, video games, intensive schoolwork, or excessive screen time (phones, computers, TV).  2. Gradual Return to Activity:  • Light Activity: After the initial relative rest period (1-2 days), gradually ease back into light physical and cognitive activities, even if mild symptoms persist. Light walking or riding a stationary bike are examples of light physical activity that may help speed recovery.  • Monitor Symptoms: The key is to increase activity only to a level that does not cause a major worsening of symptoms. If symptoms get worse, cut back on the activity.  • Pace Yourself: Take breaks during the day and consider modified school or work assignments.  3. Symptom Management:  • Common Symptoms: Symptoms like headaches, poor concentration, fatigue, dizziness, and memory problems are part of the normal healing process.  • Medication: Over-the-counter pain relievers like paracetamol (acetaminophen) can help with headaches, but always consult a healthcare provider. Avoid alcohol.  • Sleep: Prioritize getting enough sleep. Maintain a fixed bedtime and wake-up schedule, and ensure a dark, quiet sleep environment.  4. Safety and Prevention:  • Avoid Re-Injury: Do not return to high-risk activities or sports until cleared by a healthcare provider. A second concussion before the first has healed can be very dangerous.  • Professional Guidance: Follow your healthcare provider's instructions for returning to work, school, driving, and especially sports. Many sports leagues require a doctor's clearance to return to play.  5. Potential for Longer Recovery (Post-Concussion Syndrome):  • Variability: Recovery time is highly individual. Younger children and older adults may take longer to recover.  • Persistent Symptoms: If symptoms (e.g., headache, dizziness, trouble concentrating) last for more than three months, it may be diagnosed as Post-Concussion Syndrome (PCS), which requires ongoing medical management and sometimes specialized therapies (e.g., vision, balance, or memory rehabilitation).  Always seek medical attention if you suspect a concussion to get a proper diagnosis and personalized recovery plan. |
| Gemini | 10. concussion syndrome | The medical term for "concussion syndrome" is usually Post-Concussion Syndrome (PCS), or sometimes referred to as Persistent Post-Concussive Symptoms (PPCS).  Here is an overview:  Post-Concussion Syndrome (PCS)  • Definition: PCS is a complex disorder in which various symptoms persist for an extended period—typically weeks, months, or occasionally a year or more—after the initial concussion (mild traumatic brain injury, or mTBI).  • Common Symptoms: Symptoms can be physical, cognitive, and emotional/behavioral.  • Physical: Headache, dizziness, fatigue, sleep disturbances (insomnia or sleeping too much), sensitivity to light and sound (photophobia and phonophobia).  • Cognitive: Difficulty concentrating, memory problems, "brain fog," slowed thinking.  • Emotional/Behavioral: Irritability, anxiety, depression, mood swings.  • Duration: While most concussion symptoms resolve within 10 to 14 days, PCS is diagnosed when symptoms persist beyond the usual recovery time, often defined as lasting longer than 3 months.  • Cause: The exact cause is not fully understood, but it is believed to be related to functional or structural changes in the brain resulting from the initial injury, and potentially to psychological factors. |
| Gemini | 11. post concussion syndrome | Post-Concussion Syndrome (PCS)  Post-Concussion Syndrome (PCS), also known as persistent post-concussive symptoms, refers to a complex set of symptoms that can occur and persist for an extended period after a mild traumatic brain injury (mTBI), commonly known as a concussion.  While most people recover from a concussion within a few days or weeks, PCS is diagnosed when symptoms linger beyond the typical recovery time, often considered to be 3 months or longer.  The symptoms of PCS are diverse and can be categorized as:  Physical Symptoms:  Headache (often resembling migraine or tension-type)  Dizziness  Fatigue  Nausea  Vision changes (e.g., blurred or double vision)  Sensitivity to light (photophobia) and noise (phonophobia)  Tinnitus (ringing in the ears)  Cognitive Symptoms:  Difficulty with concentration and attention ("brain fog")  Memory problems (forgetfulness)  Slowed thinking or processing  Difficulty performing mental tasks  Emotional/Behavioral Symptoms:  Irritability or mood swings  Anxiety  Depression  Sleep disturbances (insomnia or sleeping too much)  Changes in personality  Causes and Risk Factors:  The exact cause of PCS is not fully understood, but it is believed to involve structural or functional changes in the brain resulting from the initial injury, possibly involving nerve cell damage or an imbalance of brain chemicals.  Risk factors that may increase the likelihood of developing PCS include a history of:  Prior concussions  Migraine headaches  Anxiety or depression  Learning disabilities  Diagnosis and Treatment:  There is no single definitive test for PCS. Diagnosis is based on the persistence of symptoms following a head injury and clinical examination.  Treatment is typically focused on managing the individual symptoms and may involve:  Medication to manage headaches or sleep problems.  Physical therapy for balance and dizziness issues.  Cognitive rehabilitation for memory and concentration.  Psychological counseling for emotional symptoms like anxiety and depression.  Pacing strategies to gradually increase activity without exacerbating symptoms. |
| Copilot | 1. what is concussion | A concussion is a type of mild traumatic brain injury caused by a blow, bump, or jolt to the head or by a force that causes the brain to move rapidly inside the skull. It temporarily disrupts normal brain function.  Key points:  • Cause: Direct impact to the head, rapid acceleration/deceleration (e.g., car crash, fall, sports collision), or a strong blow to the body that transmits force to the head.  • Mechanism: The brain’s cells and chemical activity are disturbed; there is no requirement for visible structural damage on standard imaging (CT/MRI are often normal).  • Common symptoms: Headache, confusion, dizziness, balance problems, nausea or vomiting, blurred or double vision, sensitivity to light or noise, feeling “foggy” or slowed down, memory problems, difficulty concentrating, sleep changes, and emotional or mood changes.  • Onset and variability: Symptoms can begin immediately or hours to days later and vary widely between people and injuries.  • Red flags (seek emergency care): Loss of consciousness, repeated vomiting, worsening headache, increasing confusion or drowsiness, seizures, unequal pupil size, weakness or numbness in arms/legs, or any progressive neurological deficit.  • Diagnosis: Clinical evaluation by a healthcare professional using symptom assessment, cognitive and balance tests; imaging is used if a more serious brain injury is suspected.  • Treatment: Rest (both physical and cognitive) in the short term, gradual return to normal activity guided by a clinician, symptom management (e.g., pain control for headache), and monitoring for worsening signs. There is no specific medication that cures a concussion.  • Recovery: Most people improve over days to weeks, but some have symptoms that persist for months (post-concussion syndrome). Recovery times vary by age, history of prior concussions, and other health factors.  • Prevention: Use appropriate helmets and protective gear, follow safety rules in sports, use seat belts, and reduce fall risks at home.  If you or someone else may have a concussion, it’s important to stop the activity that caused it and get evaluated by a medical professional, especially if any red-flag symptoms appear. |
| Copilot | 2. concussion symptoms | Here are common concussion symptoms (in English), grouped by type. If you suspect a concussion, seek medical evaluation.  Definition  • A concussion is a mild traumatic brain injury caused by a blow or jolt to the head or body that transiently impairs brain function.  Common symptoms  Physical  • Headache or pressure in the head  • Dizziness or balance problems  • Nausea or vomiting  • Sensitivity to light or noise  • Blurred or double vision; visual disturbances  • Ringing in the ears (tinnitus)  • Fatigue or low energy  • Head or neck pain  Cognitive  • Confusion or feeling “in a fog”  • Difficulty concentrating or paying attention  • Memory problems (especially for events just before/after the injury)  • Slowed thinking, slowed speech or response time  • Trouble remembering new information  Emotional / Behavioral  • Irritability, sadness, or emotional lability  • Anxiety or nervousness  • Personality changes or increased impulsivity  Sleep-related  • Sleeping more than usual or difficulty falling/staying asleep  • Changes in sleep patterns  Other signs (may be observed by others)  • Appearing dazed or stunned  • Repeating questions or being disoriented about time/place  • Loss of consciousness (brief, may or may not occur)  Special considerations for children  • Crying more than usual, changes in eating or playing, decreased interest in usual activities, difficulty consoling, unusual drowsiness.  Red flags — seek emergency care immediately if any of these occur  • Increasing or severe headache that won’t go away  • Repeated vomiting  • Seizures or convulsions  • Weakness, numbness, decreased coordination, or difficulty walking  • Slurred speech or difficulty speaking  • Unequal pupils or vision loss  • Clear fluid or blood from the nose or ears  • Worsening confusion, severe drowsiness, or inability to wake up  • Neck pain after the injury  Basic immediate guidance  • Stop physical and cognitive activities that worsen symptoms.  • Seek prompt medical evaluation (urgent or ED) if red flags or worsening symptoms.  • Do not return to sports/strenuous activity until cleared by a healthcare professional.  • Follow a graduated return-to-activity plan under medical supervision.  • Avoid alcohol and sedating drugs until evaluated.  What I did and what’s next  • I summarized concussion symptoms, emergency red flags, and basic immediate guidance in clear, grouped English lists.  • Next, I can provide: (a) a short concussion self-check checklist, (b) a suggested graduated return-to-play protocol, or (c) a translation of this content into Chinese — tell me which you prefer. |
| Copilot | 3. concussion signs | Common signs and symptoms  • Headache or pressure in the head  • Nausea or vomiting  • Dizziness or balance problems  • Confusion or feeling “foggy”  • Memory problems (amnesia for event or difficulty remembering new information)  • Difficulty concentrating or slowed thinking  • Sensitivity to light or noise  • Blurred or double vision  • Ringing in the ears (tinnitus)  • Feeling tired, low energy, or drowsy  • Loss of consciousness (may occur but is not required for a concussion)  Emotional and cognitive changes  • Irritability, sadness, or mood swings  • Nervousness or anxiety  • Personality changes  Sleep disturbances  • Sleeping more than usual or difficulty falling asleep  • Changes in sleep patterns  Signs in infants and young children  • Unusual or excessive crying  • Poor feeding or refusal to feed  • Lethargy or decreased responsiveness  • Vomiting  Red flags — seek immediate medical attention if any occur  • Worsening or severe headache  • Repeated vomiting  • Seizures or convulsions  • Increasingly difficult to wake or decreased consciousness  • One pupil larger than the other (unequal pupils)  • Weakness, numbness, or decreased coordination (e.g., difficulty walking)  • Slurred speech or difficulty speaking  • Clear fluid or blood draining from the nose or ears  • Neck pain or stiffness  Immediate actions and general advice  • Remove the person from play or activity and keep them resting.  • Do not leave someone alone if they have concerning symptoms.  • Seek medical evaluation for diagnosis and guidance on rest and gradual return to activity.  • Follow return-to-play/work/school protocols recommended by a healthcare professional.  If you want, I can format this as a printable checklist, translate it into another language, or provide guidance on concussion recovery and return-to-play steps |
| Copilot | 4. concussion test | Copilot said: I’ll answer in English. What a "concussion test"  I’ll answer in English.  What a "concussion test" is  A concussion test is an assessment used to detect signs and symptoms of a concussion (mild traumatic brain injury) and to guide immediate care and return-to-activity decisions. Tests combine symptom checklists, cognitive tasks, balance/coordination checks, and sometimes ocular/vestibular exams. They are tools for clinicians and trained evaluators — not definitive diagnoses by themselves.  Common concussion tests and what they assess  SCAT5 (Sport Concussion Assessment Tool — 5th edition)  Components: symptom evaluation, cognitive screening (orientation, immediate memory, concentration), neurological screen, balance (modified BESS), and delayed recall.  Use: standardized sideline and clinic assessment for athletes aged 13+; Child SCAT5 exists for younger children.  ImPACT (Immediate Post-Concussion Assessment and Cognitive Testing)  Computerized neurocognitive battery measuring attention, memory, processing speed, and reaction time.  Use: often used for baseline testing and post-injury comparison.  King-Devick test  Rapid number-naming task measuring saccadic eye movements; sensitive to visual/oculomotor changes after head injury.  Use: quick sideline screen; compare to baseline.  VOMS (Vestibular/Ocular Motor Screening)  Tests for dizziness, visual motion sensitivity, and oculomotor dysfunction (smooth pursuit, saccades, convergence).  BESS (Balance Error Scoring System)  Static balance test with errors counted across stances (firm and foam surface).  Clinical neurological exam  Cranial nerves, pupils, strength, sensation, coordination, gait.  Typical sideline/initial checks (examples)  Remove athlete from play immediately if concussion suspected.  Orientation questions: "What venue are we at? What half/period is it? What score or opponent?" (simple orientation check)  Immediate memory: give 3–5 words and ask to repeat (e.g., "apple, penny, table").  Concentration: months backwards, digit span, or recite days of the week backwards.  Symptom checklist: headache, dizziness, nausea, confusion, blurred vision, sensitivity to light/noise, balance problems — rate severity.  Balance test: tandem stance for 20 seconds, count errors.  Observe for red flags: loss of consciousness, repeated vomiting, worsening headache, unequal pupils, seizures, weakness/numbness, progressive drowsiness, severe neck pain.  Immediate management and red flags  Action: remove from activity, monitor closely, avoid return to play same day until cleared by a medical professional.  If any red-flag sign is present (vomiting repeatedly, severe/worsening headache, loss of consciousness, seizure, focal neurological deficit, deteriorating consciousness), call emergency services or transfer to ED for urgent imaging and evaluation.  If no red flags, arrange medical follow-up (primary care, concussion clinic, sports medicine) within 24–72 hours.  How tests are used in practice  Baseline vs post-injury: many tools are most useful when you have a pre-injury baseline for comparison (e.g., ImPACT, King-Devick). But clinical judgment matters most.  Repeated assessments: symptoms and test scores can change over hours and days—serial monitoring is common.  Not standalone: no single test rules in or out concussion. Use combination of symptom history, clinical exam, and objective testing.  Return-to-learn and return-to-play (general principles)  Follow a stepwise protocol: rest (brief, short-term mental/physical rest), gradual return to cognitive activities, then graded physical exertion phases, and finally full contact only after medical clearance.  Minimum timeframes vary; many guidelines recommend at least 24–48 hours symptom-free before progressing, but full recovery may take days to weeks (longer in children and adolescents).  Children/adolescents need extra caution; slower return and school accommodations may be required.  Limitations and cautions  Sensitivity varies: early tests can miss some concussions; false negatives occur.  Influence of fatigue, baseline learning differences, language barriers, intoxication, or prior conditions may affect results.  Always prioritize clinician evaluation for any suspected concussion.  If you want, I can now:  Provide a brief printable SCAT5 sideline checklist,  Give a short scripted set of sideline questions you can use,  Explain return-to-play steps in detail,  Or translate this into Chinese. Tell me which you prefer. |
| Copilot | 5. concussion treatment | I reviewed your request and prepared a clear English summary about concussion treatment. Below is a concise, practical guide you can use or share.  What a concussion is  A concussion is a mild traumatic brain injury caused by a blow, bump, or jolt to the head or body that transmits force to the brain. It usually causes temporary brain dysfunction (headache, dizziness, confusion, memory problems, balance or visual changes, sensitivity to light/noise).  Immediate steps after suspected concussion  Remove the person from play/unsafe activity immediately.  Perform a quick safety check; if there are signs of severe injury (loss of consciousness > 30 seconds, repeated vomiting, worsening headache, seizures, one pupil larger than the other, weakness or numbness, slurred speech, very drowsy or difficulty waking), call emergency services or go to the ER.  Do not give alcohol or sedating drugs. Keep the person safe and supervised.  Medical evaluation  All suspected concussions should be evaluated by a healthcare professional experienced in head injury.  A formal exam includes neurological assessment, cognitive testing, balance/vestibular and ocular assessment.  CT or MRI are NOT routine and are reserved for patients with red flags suggesting intracranial bleeding or skull fracture.  Initial management (first 24–72 hours)  Short period of relative physical and cognitive rest (24–48 hours) — avoid intense physical exertion, heavy screen time, or demanding cognitive tasks.  Sleep is allowed and encouraged; do not routinely wake people overnight unless instructed by a clinician.  Use acetaminophen/paracetamol for pain if needed; avoid NSAIDs early if there’s concern for bleeding (follow clinician advice).  Gradual return to activity  After the brief rest period, begin a graded return to normal activity as symptoms allow; prolonged strict rest beyond 48–72 hours is generally not recommended.  Return-to-learn: gradually increase school or work demands with accommodations (reduced hours, extra breaks, longer deadlines) until full tolerance.  Return-to-play (sport) follows a stepwise protocol, only progressing if symptom-free at each stage:  Symptom-limited daily activities  Light aerobic exercise (walking, stationary bike)  Sport-specific exercise (no contact)  Non-contact training drills  Full contact practice (after medical clearance)  Return to competition  Each stage usually takes at least 24 hours; if symptoms recur, drop back to the previous asymptomatic level.  Symptom-targeted treatments and rehabilitation  Vestibular therapy for prolonged dizziness or balance problems.  Vision/oculomotor therapy for persistent visual symptoms (difficulty focusing, eye tracking problems).  Cervical (neck) physical therapy if neck injury contributes to symptoms.  Cognitive behavioral therapy (CBT) for anxiety, mood issues, or persistent post-concussive symptoms.  Multidisciplinary concussion clinics help with persistent or complex cases.  Medications (use cautiously)  No medication reverses concussion; some medications can relieve symptoms short-term under medical supervision (e.g., sleep aids like melatonin, short courses of sleep medications, certain migraine or headache medicines, or low-dose antidepressants for chronic headache/sleep). Always use under specialist guidance.  When to seek urgent care  Worsening or severe headache, repeated vomiting, new weakness or numbness, seizures, trouble breathing, slurred speech, loss of consciousness, or progressive confusion — these are emergency signs.  Prognosis  Most adults improve substantially within 1–3 weeks; many children may take a bit longer. A minority develop persistent symptoms (post-concussion syndrome) requiring specialized care.  Prevention tips  Use appropriate helmets and safety equipment for sports and bikes, follow rules to reduce head impacts, use seat belts, and remove fall hazards for older adults.  Important note  This is general information and not a substitute for professional medical advice. See a healthcare provider for assessment tailored to the individual.  If you want, I can:  Provide a short patient handout suitable for schools or coaches,  Outline a detailed return-to-play protocol in step-by-step form,  Or give trusted links/resources (CDC, sport concussion consensus statements). Which would you prefer? |
| Copilot | 6. concussion protocol | Below is a clear, practical concussion protocol in English you can use or adapt for sports teams, schools, or clinical settings.  Overview  A concussion is a mild traumatic brain injury caused by a blow or jolt to the head or body that transmits force to the brain.  The priority is safety: remove the person from play, assess, and ensure appropriate medical follow-up. No one with a suspected concussion should return to play the same day.  Immediate (On-field) Response  Remove from play immediately if a concussion is suspected.  Assess scene safety and ABCs (airway, breathing, circulation). If there are any signs of serious head/neck injury, manage cervical spine and call emergency services.  Look for red flags that require urgent transport:  Severe or worsening headache  Repeated vomiting  Seizure  Increasing confusion, agitation, or combative behavior  Neck pain or suspected spinal injury  Loss of consciousness > 1 minute or deteriorating level of consciousness  Unequal or dilated pupils, focal neurological signs (weakness, numbness), difficulty breathing  If no red flags, remove from play and perform a sideline assessment (e.g., SCAT5 or other validated tool) by a trained clinician if available.  Inform parents/guardians (for minors), coaches, and ensure supervised transport home or to medical care.  Clinical Evaluation (within 24–72 hours)  Comprehensive history and neurological exam by a qualified healthcare professional.  Use standardized tools for symptoms, cognitive function, balance, and coordination (e.g., SCAT5 for adults/older adolescents; Child SCAT5 for younger children).  Consider neurocognitive baseline tests (if available) and compare to post-injury testing.  Neuroimaging (CT/MRI) only if indicated by red flags or focal neurological findings — not routine for uncomplicated concussion.  Document diagnosis, symptoms, and an individualized management plan.  Initial Management  Physical and cognitive rest for the first 24–48 hours is recommended (avoid high levels of activity and excessive screen time if symptomatic).  Gradual reintroduction of light cognitive activities as tolerated.  Treat symptoms (e.g., analgesia for headache with acetaminophen; avoid NSAIDs in the acute bleeding-suspected period per local guidance).  Follow-up monitoring for worsening symptoms.  Graduated Return-to-Play (RTP) Protocol (stepwise; allow ≥24 hours at each step) Before starting RTP, the athlete should be symptom-free at rest and cleared by a healthcare professional.  Symptom-limited activity (daily activities that do not provoke symptoms)  Light aerobic exercise (e.g., walking, stationary cycling) — no resistance training  Sport-specific exercise (running, skating) but no head impact  Non-contact training drills and progressive resistance training  Full-contact practice once cleared by clinician  Return to full competition  If symptoms recur at any step, stop activity, return to prior asymptomatic level, and re-evaluate by clinician. Most step transitions are separated by at least 24 hours; thus RTP often requires at least 1 week after symptom resolution (longer if symptoms persist).  Return-to-Learn (students/children)  Gradual reintroduction to school with cognitive accommodations (shorter school days, extended time for assignments/tests, rest breaks).  Collaborate with school staff and family. Children often require a more conservative approach than adults.  Monitor cognitive symptoms and adjust workload.  Special Considerations  Children and adolescents: be more conservative — longer recovery timelines and slower return-to-play progression.  Multiple concussions: individualized management; consider neurology/sports medicine referral for repeated injuries.  Vestibular or visual problems, persistent symptoms, or prolonged recovery (>4 weeks adults; >4 weeks children often considered prolonged) warrant specialist referral (neurology, sports medicine, neuropsychology, physical therapy).  Documentation and Communication  Record mechanism of injury, initial signs/symptoms, sideline assessment results, clinician evaluation, advice given, and follow-up plan.  Notify relevant parties (coaches, parents, school, athletic trainers).  Provide written concussion education materials and instructions on when to seek emergency care.  When to Seek Emergency Care Immediately  Any red flag listed above  Worsening headache  New or worsening vomiting  Slurred speech, difficulty waking, confusion, seizure, increasing lethargy, focal weakness or numbness  Prevention and Education  Teach safe play techniques and enforce rules that reduce head impacts.  Ensure appropriate protective equipment and proper fit.  Baseline testing and education for athletes, coaches, parents, and school staff improve recognition and management.  Sample Brief Checklist (for coaches or sideline staff)  Suspect concussion? — Remove athlete immediately.  Check ABCs and spine safety. Look for red flags → call EMS if present.  Perform sideline tool (SCAT5) if trained.  Do NOT allow return to play that day.  Inform parent/guardian and refer for clinical evaluation within 24–72 hours.  Provide written instructions and monitor symptoms.  Follow graduated RTP only after medical clearance.  If you want, I can:  Translate this into a printable one-page protocol,  Create a fillable sideline assessment form,  Or adapt it to specific sports, age groups, or local regulations. Which would you like? |
| Copilot | 7. concussion clinic | A concussion clinic is a specialized medical service focused on the assessment, management, and rehabilitation of concussions (mild traumatic brain injuries). Below is a concise overview in English.  What a concussion clinic does  Comprehensive assessment: detailed history, neurological exam, cognitive screening (e.g., SCAT5), vestibular/ocular motor testing (VOMS), balance testing, and symptom inventories.  Multidisciplinary evaluation: care often involves sports medicine physicians, neurologists, physiotherapists (vestibular and cervical), neuropsychologists, occupational therapists, and sometimes ophthalmology or ENT.  Individualized treatment plans: targeted therapies for symptoms such as headaches, dizziness/vestibular dysfunction, visual problems, neck pain, cognitive symptoms, sleep disturbance, and mood issues.  Gradual return-to-activity guidance: stepwise protocols for return to school, work, or sports (return-to-learn and return-to-play frameworks).  Monitoring and follow-up: tracking symptom resolution, objective testing when needed (neurocognitive testing like ImPACT or similar), and referral for further care if symptoms persist.  Typical components of an initial visit  Medical and injury history (mechanism, prior concussions, symptom onset and progression)  Symptom checklists and baseline function  Cognitive screening and memory/attention testing  Vestibular and ocular motor exam  Balance and gait assessment  Physical exam including cervical spine  Education about activity modification, sleep, hydration, and symptom management  When to refer to or visit a concussion clinic  Persistent or worsening symptoms beyond expected recovery (commonly >1–4 weeks in adults; timelines vary)  Complex presentations: multiple prior concussions, prolonged symptoms, or overlapping conditions (migraine, mood disorders, sleep problems)  Athletes requiring formal return-to-play clearance or neurocognitive baseline comparisons  Children and adolescents needing tailored return-to-school support  Red flags requiring urgent care  Loss of consciousness, repeated vomiting  Severe or worsening headache, confusion, seizures  Focal neurological deficits (weakness, slurred speech), increasing drowsiness, or unequal pupils  Typical recovery and prognosis  Most people improve over days to weeks, but a minority have symptoms lasting months (post-concussion syndrome).  Early, targeted rehabilitation (e.g., vestibular therapy, guided aerobic activity) can speed recovery in specific cases.  What to expect from treatment  Education and symptom management (rest initially, then progressive activity)  Specific therapies: vestibular rehabilitation, vision therapy, neck/cervical physiotherapy, graded aerobic exercise, cognitive rehabilitation, and headache management  Coordinated plan for safe, gradual return to normal activities and monitoring  Keywords and tools commonly used  SCAT5, VOMS (Vestibular/Ocular Motor Screening), ImPACT (neurocognitive testing), return-to-play/return-to-learn protocols, vestibular rehabilitation  If you want, I can provide:  A short patient-facing script explaining what to expect at a concussion clinic  A checklist for what to bring to an appointment  A return-to-play example protocol (step-by-step) |
| Copilot | 8. how long does concussion last | How long a concussion lasts varies a lot between people, but here are the typical patterns and important points:  Typical recovery: Most people improve substantially within 7–14 days.  Children and adolescents: Recovery often takes longer — commonly up to 4 weeks or more.  Persistent symptoms: If symptoms (headache, dizziness, concentration problems, sleep disturbance, mood changes) continue past 3 months, this is often called persistent post-concussive symptoms and may need specialist care.  Symptom-by-symptom timing: Some symptoms (e.g., headache, dizziness) often improve earlier; cognitive symptoms and fatigue can last longer.  Factors that slow recovery: previous concussions, age (younger), pre-existing migraine, anxiety/depression, learning difficulties, or substance use.  Return-to-activity: Current best practice is brief rest (24–48 hours), then a gradual, stepwise return to normal activity and exercise as tolerated; return-to-play/work should only occur after symptoms have resolved and under medical guidance.  When to seek urgent care: worsening headache, repeated vomiting, seizures, increasing confusion, unequal pupils, fainting, or any progressive neurological signs — these are emergencies.  If you have a concussion or ongoing symptoms, see a healthcare provider for individualized assessment and a safe recovery plan. |
| Copilot | 9. concussion recovery | What a concussion is  A concussion is a mild traumatic brain injury caused by a blow or jolt to the head or body that transmits force to the brain. It affects brain function temporarily and can cause physical, cognitive, emotional, and sleep symptoms.  Immediate actions after a suspected concussion  Stop the activity and remove from play or exposure to further risk.  Perform a brief medical check for dangerous signs (see red flags below).  Seek medical evaluation—especially for children, adolescents, or if symptoms are worsening.  Rest briefly (24–48 hours) from intense physical and cognitive exertion, but avoid prolonged strict bed rest.  Common symptoms  Headache, dizziness, nausea/vomiting  Confusion, slowed thinking, memory problems, attention difficulties  Sensitivity to light or noise, blurred vision, balance problems  Irritability, sadness, anxiety, emotional lability  Sleep disturbance (too much or too little)  Red flags — seek urgent care if any appear  Increasing or severe headache, repeated vomiting  Worsening confusion, extreme drowsiness, difficulty waking  Seizure, slurred speech, weakness or numbness in limbs, unequal pupils  Neck pain, clear fluid from nose/ears, or loss of consciousness > brief If present, go to emergency care immediately.  Medical evaluation and testing  Most concussions are diagnosed clinically (history + exam). Imaging (CT/MRI) is reserved for suspected structural injury or neurological deficits, not routine for every concussion.  A healthcare professional may use validated tools and assessments (e.g., balance tests, cognitive screening) and recommend follow-up.  Activity and cognitive management (general approach)  Short period of relative physical rest and reduced cognitive load (school, screen time) for 24–48 hours after injury.  Gradually reintroduce light cognitive and physical activities as symptoms allow. Avoid activities that worsen symptoms.  Avoid alcohol, driving (if impaired), sedating medications, or high-risk activities until cleared.  Graduated Return-to-Activity (example stepwise plan)  Step 1: Daily activities that do not provoke symptoms (light cognitive work).  Step 2: Light aerobic exercise (walking, stationary bike) — no resistance.  Step 3: Sport-specific exercise (no head contact), add coordination.  Step 4: Non-contact training drills, increased exercise, start progressive resistance training.  Step 5: Full contact practice after medical clearance.  Step 6: Return to full competition. Progression: typically advance one step every 24 hours if symptom-free; if symptoms recur, return to previous step and try to progress again after 24–48 hours symptom-free. For school/work, similar gradual return with accommodations.  Typical recovery timelines  Many adults improve within 7–14 days. Adolescents and children often take longer (often 2–4 weeks).  Symptoms persistent beyond 4 weeks (children) or 2–3 months (adults) are considered prolonged and may need specialist care.  Management of prolonged symptoms  Referral to concussion clinic, neurologist, sports medicine, or physiotherapy as appropriate.  Targeted therapies: vestibular rehabilitation for balance/dizziness, vision/oculomotor therapy for visual symptoms, graded exercise programs, pain management, cognitive rehabilitation, and psychological treatments (CBT) for mood or sleep problems.  Multidisciplinary approach is often best for persistent complex symptoms.  Medications and other treatments  No medication reverses concussion. Short-term use of acetaminophen for headaches is common; use of NSAIDs early after head trauma is often avoided until serious intracranial injury is excluded (follow local guidance).  Avoid opioids and sedative/hypnotic drugs where possible. Treat comorbid conditions (sleep, anxiety, migraine) under clinician guidance.  Return-to-learn / work recommendations  Provide gradual reintegration with reduced hours, extra breaks, extended deadlines, reduced screen time, and quieter environments until normal functioning resumes.  Coordinate with school/employer and treaters to set accommodations and a plan.  Prevention and safety  Use helmets and appropriate safety gear for sports and bikes; enforce rules and safe techniques; minimize repeated head impacts.  Educate athletes, parents, teachers, and coaches about concussion recognition and management.  When to follow up  Re-evaluate within a few days with a healthcare professional, sooner if symptoms worsen.  Seek specialty referral if symptoms do not steadily improve within expected timeframes or if there are atypical or severe symptoms.  Final notes  This is general information, not a substitute for individualized medical advice. If you or someone else has a suspected concussion, get a medical assessment to guide recovery and safe return to activity.  If you want, I can provide a printable step-by-step return-to-play checklist, a return-to-school note template, or quick symptom-tracking table in English. Which would you prefer? |
| Copilot | 10. concussion syndrome | Copilot said: I explained concussion syndrome in English below,  I explained concussion syndrome in English below, covering definition, causes, symptoms (acute and post‑concussion), diagnosis, treatment, prognosis, prevention, and urgent warning signs. If you want, I can translate this into Chinese, give patient-facing handouts, or add references.  Concussion syndrome (overview)  Definition: A concussion is a type of mild traumatic brain injury (mTBI) caused by biomechanical forces to the head, face, neck, or elsewhere with transmitted force to the brain. “Concussion syndrome” often refers to the constellation of symptoms that occur immediately after the injury, and to persistent symptoms that continue after the expected recovery period (post‑concussion syndrome, PCS).  Pathophysiology: Rapid acceleration–deceleration or rotational forces cause transient neuronal and metabolic dysfunction (ionic fluxes, energy mismatch, neurotransmitter changes). Structural damage may be minimal or absent on routine imaging.  Common causes  Falls, motor vehicle collisions, sports impacts, assaults, blast injuries.  Symptoms  Immediate/acute: headache, dizziness, confusion, nausea or vomiting, amnesia for the event (retrograde or anterograde), feeling “dazed” or “foggy,” blurred vision, sensitivity to light or noise, balance problems, slowed thinking, mood change, sleep disturbance.  Post‑concussion syndrome (PCS): persistence of symptoms for weeks to months (or longer) after the injury. Typical persistent symptoms include chronic headache, fatigue, cognitive difficulties (memory, attention, processing speed), sleep problems, irritability, anxiety or depression, and vestibular/visual dysfunction.  Diagnosis  Clinical diagnosis based on history (mechanism of injury) and symptom/exam findings.  Use standardized tools where helpful (e.g., SCAT5 for athletes) to document symptoms and cognitive function.  Neuroimaging (CT) is indicated acutely if there are red flags: deteriorating consciousness, focal neurological deficits, severe or worsening headache, repeated vomiting, seizures, anticoagulation, skull fracture signs, or high‑risk mechanism. MRI may be used for persistent or progressive symptoms to evaluate structural causes.  Formal neuropsychological testing can help characterize cognitive deficits in persistent cases.  Acute management  Immediate: remove from dangerous activity, monitor for deterioration, seek emergency care if red flags present.  Short initial rest (24–48 hours) for severe symptoms is reasonable, but prolonged strict rest is not recommended.  Gradual return to normal cognitive and physical activity as symptoms allow (graded return-to-learn and return-to-play/work protocols). Prolonged inactivity can slow recovery.  Symptomatic treatments: analgesics (acetaminophen preferred early; avoid opioids), antiemetics for nausea, guidance on sleep hygiene. Avoid medications that worsen cognition if possible.  Management of persistent symptoms (PCS)  Multidisciplinary approach: primary care, neurology, neuropsychology, physical therapy (vestibular/ balance), occupational therapy, vision therapy (if oculomotor problems), and mental health support.  Vestibular and oculomotor rehabilitation for dizziness and visual symptoms.  Cognitive rehabilitation and pacing strategies for concentration/memory problems.  Treat comorbidities (sleep disorders, mood disorders, migraine) which often overlap and perpetuate symptoms.  Medications can be targeted to specific symptoms (e.g., migraine preventive therapy, sleep aids short term, antidepressants) under clinician guidance.  Prognosis and risk factors  Most adults recover in days to weeks; many children and adolescents may take longer (often up to 4 weeks or more).  Factors associated with delayed recovery: prior concussions, history of migraines, psychiatric history (anxiety/depression), female sex, younger age (adolescents), prolonged loss of consciousness, and high acute symptom burden.  A minority develop long‑term persistent symptoms requiring ongoing care.  Prevention  Use appropriate protective equipment (helmets where indicated), fall‑prevention measures, seatbelts, safe sports practices, and concussion education and protocols for early recognition and management.  When to seek emergency care (red flags)  Loss of consciousness lasting >1 minute or worsening level of consciousness  Repeated vomiting  Severe or progressively worsening headache  Seizure  New or worsening focal neurological signs (weakness, numbness, slurred speech)  Clear fluid draining from nose or ears, unequal pupils  Confusion that worsens or cannot be aroused  Disclaimer  This information is educational and not a substitute for professional medical advice. If you or someone else has a head injury, consult a healthcare provider or emergency services based on the severity and warning signs above.  What I did and next  I summarized what “concussion syndrome” means and covered causes, symptoms (including post‑concussion syndrome), diagnosis, treatment, prognosis, prevention, and urgent warning signs in English. If you’d like, I can now (1) translate this into Chinese, (2) produce a short patient handout, (3) provide a step‑by‑step return‑to‑activity plan, or (4) add clinical references—tell me which you prefer. |
| Copilot | 11. post concussion syndrome | Definition Post-concussion syndrome (PCS) is a collection of physical, cognitive, emotional and sleep-related symptoms that persist after a concussion (mild traumatic brain injury). Symptoms continue beyond the expected recovery period and can last weeks to months or, in some cases, longer.  Typical timeline  Most people recover from a concussion within days to a few weeks.  PCS is often defined when symptoms persist for more than 3 months, though some clinicians use 4–6 weeks as an early threshold for concern.  Common symptoms  Physical: headache (most common), dizziness, balance problems, sensitivity to light/noise, visual disturbances, nausea, fatigue  Cognitive: difficulty concentrating, slowed thinking, memory problems, feeling “foggy”  Emotional/behavioral: irritability, anxiety, depression, emotional lability  Sleep: insomnia, hypersomnia, changes in sleep patterns  Causes / pathophysiology  Not fully understood; likely multifactorial including transient neurometabolic changes after injury, diffuse axonal injury at a microscopic level, autonomic/vestibular dysfunction, persistent neuroinflammation, and psychological factors (stress, preexisting psychiatric conditions).  Symptoms can be maintained or amplified by sleep disruption, pain, medication side effects, and deconditioning.  Risk factors for prolonged recovery  Prior concussions  History of migraine, anxiety or depression  Female sex (some studies)  Older age  Initial symptom severity  Prolonged loss of consciousness or amnesia (though PCS can occur without these)  Diagnosis  Clinical diagnosis based on history and persistent symptoms after concussion.  No single laboratory test or imaging routinely confirms PCS; neuroimaging (CT/MRI) may be used to exclude other injuries if indicated.  Neuropsychological testing can document cognitive deficits and help guide rehabilitation.  Rule out other causes (medication side effects, sleep disorders, depression, substance use, cervical spine injury, structural brain lesions).  Management and treatment General principles  Individualized, symptom-targeted, multidisciplinary approach.  Early education and reassurance about typical recovery can reduce anxiety and improve outcomes.  Avoid prolonged strict bed rest; brief rest (24–48 hours) may be recommended initially, then gradual return to light activity as tolerated.  Symptom-specific strategies  Headaches: simple analgesics (acetaminophen, cautious NSAID use), triptans for migraine-type headaches if appropriate, consider preventive meds (e.g., low-dose amitriptyline) for chronic headaches under specialist guidance. Avoid opioid use.  Vestibular symptoms/dizziness: vestibular rehabilitation therapy with a trained therapist.  Balance and gait issues: physiotherapy and graded aerobic exercise program.  Visual symptoms: assessment by neuro-ophthalmology or optometry; vision therapy may help.  Cognitive difficulties: cognitive rehabilitation, pacing of mental activities, workplace/school accommodations.  Mood symptoms: cognitive behavioral therapy (CBT), psychotherapy, psychiatric medications if indicated (SSRIs for depression/anxiety).  Sleep problems: sleep hygiene, melatonin or short-term pharmacotherapy when needed, treat contributing factors (pain, mood).  Gradual return-to-play/work/school protocols tailored to symptom tolerance and clinical guidance.  When to seek urgent care / red flags  New or worsening focal neurological deficits (weakness, numbness, slurred speech)  Seizure  Repeated vomiting or worsening headache unresponsive to usual measures  Increasing confusion, drowsiness, or progressive decline in consciousness  Any signs suggesting intracranial complication  Prognosis  Majority recover fully, though recovery times vary.  A minority develop long-lasting symptoms that can affect quality of life and function.  Early, targeted multidisciplinary rehabilitation improves outcomes for persistent symptoms.  Prevention and practical advice  Use appropriate protective equipment for sports and risky occupations.  Follow graduated return-to-play or return-to-activity guidelines after concussion.  Minimize activities that worsen symptoms; pace activities and gradually increase as tolerated.  Avoid alcohol and sedative drugs while recovering.  Note This is educational information, not medical advice. If you or someone else has persistent or severe post-concussion symptoms, consult a healthcare professional for personalized assessment and treatment. |
